# Structural Modeling of the Treponema pallidum Outer Membrane Protein Repertoire: a Road Map for Deconvolution of Syphilis Pathogenesis and Development of a Syphilis Vaccine

**DOI:** 10.1128/JB.00082-21

**Published:** 2021-07-08

**Authors:** Kelly L. Hawley, Jairo M. Montezuma-Rusca, Kristina N. Delgado, Navreeta Singh, Vladimir N. Uversky, Melissa J. Caimano, Justin D. Radolf, Amit Luthra

**Affiliations:** a Department of Pediatrics, UConn Health, Farmington, Connecticut, USA; b Division of Infectious Diseases and Immunology, Connecticut Children’s, Hartford, Connecticut, USA; c Department of Medicine, UConn Health, Farmington, Connecticut, USA; d Division of Infectious Diseases, UConn Health, Farmington, Connecticut, USA; e Department of Molecular Medicine, Morsani College of Medicine, University of South Florida, Tampa, Florida, USA; f Department of Molecular Biology and Biophysics, UConn Health, Farmington, Connecticut, USA; g Department of Genetics and Genome Sciences, UConn Health, Farmington, Connecticut, USA; h Department of Immunology, UConn Health, Farmington, Connecticut, USA; NCBI, NLM, National Institutes of Health

**Keywords:** syphilis, *Treponema pallidum*, bioinformatics, outer membrane proteins, structural biology, vaccines

## Abstract

Treponema pallidum, an obligate human pathogen, has an outer membrane (OM) whose physical properties, ultrastructure, and composition differ markedly from those of phylogenetically distant Gram-negative bacteria. We developed structural models for the outer membrane protein (OMP) repertoire (OMPeome) of T. pallidum Nichols using solved Gram-negative structures, computational tools, and small-angle X-ray scattering (SAXS) of selected recombinant periplasmic domains. The T. pallidum “OMPeome” harbors two “stand-alone” proteins (BamA and LptD) involved in OM biogenesis and four paralogous families involved in the influx/efflux of small molecules: 8-stranded β-barrels, long-chain-fatty-acid transporters (FadLs), OM factors (OMFs) for efflux pumps, and T. pallidum repeat proteins (Tprs). BamA (TP0326), the central component of a β-barrel assembly machine (BAM)/translocation and assembly module (TAM) hybrid, possesses a highly flexible polypeptide-transport-associated (POTRA) 1-5 arm predicted to interact with TamB (TP0325). TP0515, an LptD ortholog, contains a novel, unstructured C-terminal domain that models inside the β-barrel. T. pallidum has four 8-stranded β-barrels, each containing positively charged extracellular loops that could contribute to pathogenesis. Three of five FadL-like orthologs have a novel α-helical, presumptively periplasmic C-terminal extension. SAXS and structural modeling further supported the bipartite membrane topology and tridomain architecture of full-length members of the Tpr family. T. pallidum’s two efflux pumps presumably extrude noxious small molecules via four coexpressed OMFs with variably charged tunnels. For BamA, LptD, and OMFs, we modeled the molecular machines that deliver their substrates into the OM or external milieu. The spirochete’s extended families of OM transporters collectively confer a broad capacity for nutrient uptake. The models also furnish a structural road map for vaccine development.

**IMPORTANCE** The unusual outer membrane (OM) of T. pallidum, the syphilis spirochete, is the ultrastructural basis for its well-recognized capacity for invasiveness, immune evasion, and persistence. In recent years, we have made considerable progress in identifying T. pallidum’s repertoire of OMPs. Here, we developed three-dimensional (3D) models for the T. pallidum Nichols OMPeome using structural modeling, bioinformatics, and solution scattering. The OM contains three families of OMP transporters, an OMP family involved in the extrusion of noxious molecules, and two “stand-alone” proteins involved in OM biogenesis. This work represents a major advance toward elucidating host-pathogen interactions during syphilis; understanding how T. pallidum, an extreme auxotroph, obtains a wide array of biomolecules from its obligate human host; and developing a vaccine with global efficacy.

## INTRODUCTION

Syphilis is a multistage, sexually transmitted infection renowned for its protean clinical manifestations and protracted course (1, 2). Since the start of the new millennium, the disease has undergone a dramatic resurgence in the United States, particularly among men who have sex with men (3), in addition to posing an ongoing global health threat to at-risk populations in resource-poor nations (4). These alarming trends underscore the urgent need for a vaccine with global efficacy (4). The complex natural history of syphilis reflects the invasiveness, immunoevasiveness, and inflammatory potential of its etiological agent, the pathogenic spirochete Treponema pallidum subsp. *pallidum* (5, 6). As an extreme auxotroph, T. pallidum must obtain a vast array of biomolecules from its obligate human host (7, 8). To support its distinct parasitic lifestyle (6), T. pallidum has evolved an outer membrane (OM) (see Table S1 in the supplemental material, which defines all abbreviations used in this document) whose physical properties, ultrastructure, and composition differ markedly from those of phylogenetically distant Gram-negative bacteria (9–12). The T. pallidum OM is a fragile lipid bilayer lacking lipopolysaccharide (LPS) (7) with a much lower density of membrane-spanning proteins than its Gram-negative counterparts (10, 11). The OM’s paucity of surface-exposed pathogen-associated molecular patterns and membrane-spanning proteins is the ultrastructural basis for the syphilis spirochete’s remarkable capacity for persistence and immune evasion, attributes that have earned it the designation “stealth pathogen” (6).

The Gram-negative OM is an asymmetric lipid bilayer consisting of an inner (periplasmic) leaflet of glycerophospholipids and an outer leaflet of LPS, the highly inflammatory glycolipid that establishes the permeability barrier (13). In disease-causing organisms, the OM constitutes the host-pathogen interface, serving adhesive, invasive, and immunoevasive functions integral to a bacterium’s parasitic program (14–16). The structure also houses the surface-exposed targets for protective antibodies elicited during infection or by immunization (17–20) and, therefore, is the focus of vaccine development. A characteristic feature of this unusual bilayer is its high density of integral membrane proteins that, with rare exceptions (21), adopt the versatile β-barrel conformation in which an even number of antiparallel, amphipathic β-strands circularize to form a closed structure, typically with a central channel (22, 23). Bridging adjacent strands on the external side of the OM are large extracellular loops (ECLs) that mediate interactions with host cells and tissue components (24–26) and contain B-cell epitopes (BCEs) subject to sequence/antigenic variation due to host immune pressure (27–30). Gram-negative β-barrel proteins serve transport-related functions that contribute to the maintenance of cellular homeostasis. General porins are abundant, trimeric aqueous channels for the passive diffusion of small, water-soluble molecules (31, 32), while low-abundance, energy-coupled, monomeric transporters serve as substrate-specific, ligand-gated channels for the uptake of micronutrients such as iron, nickel, and vitamin B_12_ (33). Gram-negative bacteria also express OM transporters that enable growth on alternative carbon sources, such as maltodextrins (LamB) and fatty acids (FadL) (34, 35). BamA and LptD are the terminal β-barrel components of the molecular machines that insert nascent outer membrane proteins (OMPs) and LPS, respectively, into the OM bilayer (36–38). The OMs of Gram-negative bacteria also contain a considerable number of proteins that form 8- to 10-stranded monomeric β-barrel structures that are implicated in the transport of small molecules and cell adhesion (39, 40). In addition to shuttling molecules into the cell, Gram-negative bacteria need to expel exogenous and endogenous toxins. They achieve this by coupling cytoplasmic membrane (CM)-associated efflux pumps to an interesting variation on the β-barrel theme: trimeric outer membrane factors (OMFs) in which each monomer contributes 4 β-strands to complete a single 12-stranded β-barrel (41, 42).

Among the many factors hampering the molecular characterization of T. pallidum strain Nichols rare OMPs was their lack of sequence relatedness to prototypical OMPs of Gram-negative bacteria (11). To overcome this hurdle, we mined the T. pallidum genome for proteins predicted to form β-barrels (6, 43), the hallmark conformation of OMPs in double-membrane organisms (22, 23), and subsequently demonstrated that selected candidate OMPs, in fact, adopt this conformation *in vitro* and are surface exposed *in vivo* (44, 45). The expansion of OMP structures in the RCSB PDB database (www.rcsb.org), coupled with newly developed structural algorithms (46–48), has enabled us to begin to generate three-dimensional (3D) models for candidate OMPs previously identified by secondary structure predictions (11).

For the iteration of the T. pallidum (Nichols) OMP repertoire (OMPeome) described here, we refined our structural models using solved structures as the templates, an expanded battery of computational and bioinformatic tools, recently published experimental data, and small-angle X-ray scattering (SAXS) analysis of selected recombinant aqueous soluble periplasmic domains. Our analyses revealed that, in addition to the BamA and LptD “stand-alone” proteins with novel structural features, the spirochete’s OM contains extended families of transporters and efflux pump OMFs that presumably endow the organism with a broad capacity for nutrient uptake and molecule extrusion. For BamA, LptD, and efflux pump OMFs, we identified and modeled the components of the cognate molecular machines that enable these OMPs to deliver their unique cargos into the OM bilayer or the external milieu. SAXS provided new insight into the function of T. pallidum’s β-barrel assembly machine (BAM) apparatus and additional biophysical evidence for the bipartite membrane topology of full-length members of the T. pallidum repeat (Tpr) family. Collectively, the 3D models reveal that T. pallidum has evolved a unique repertoire of OMPs to address its physiological needs while meeting the demands of stealth pathogenicity. Strategies to elicit protective antibodies by immunization with recombinant treponemal proteins require detailed knowledge of the molecular architecture of the spirochete’s OM as well as the membrane topology and structure of candidate vaccinogens. Thus, the models described here also provide a structural road map for the development of a broadly protective vaccine for a disease that has afflicted humankind for centuries.

## RESULTS AND DISCUSSION

### T. pallidum BamA (TP0326) possesses a divergent β-barrel domain and a highly flexible POTRA arm.

**FIG 1:**
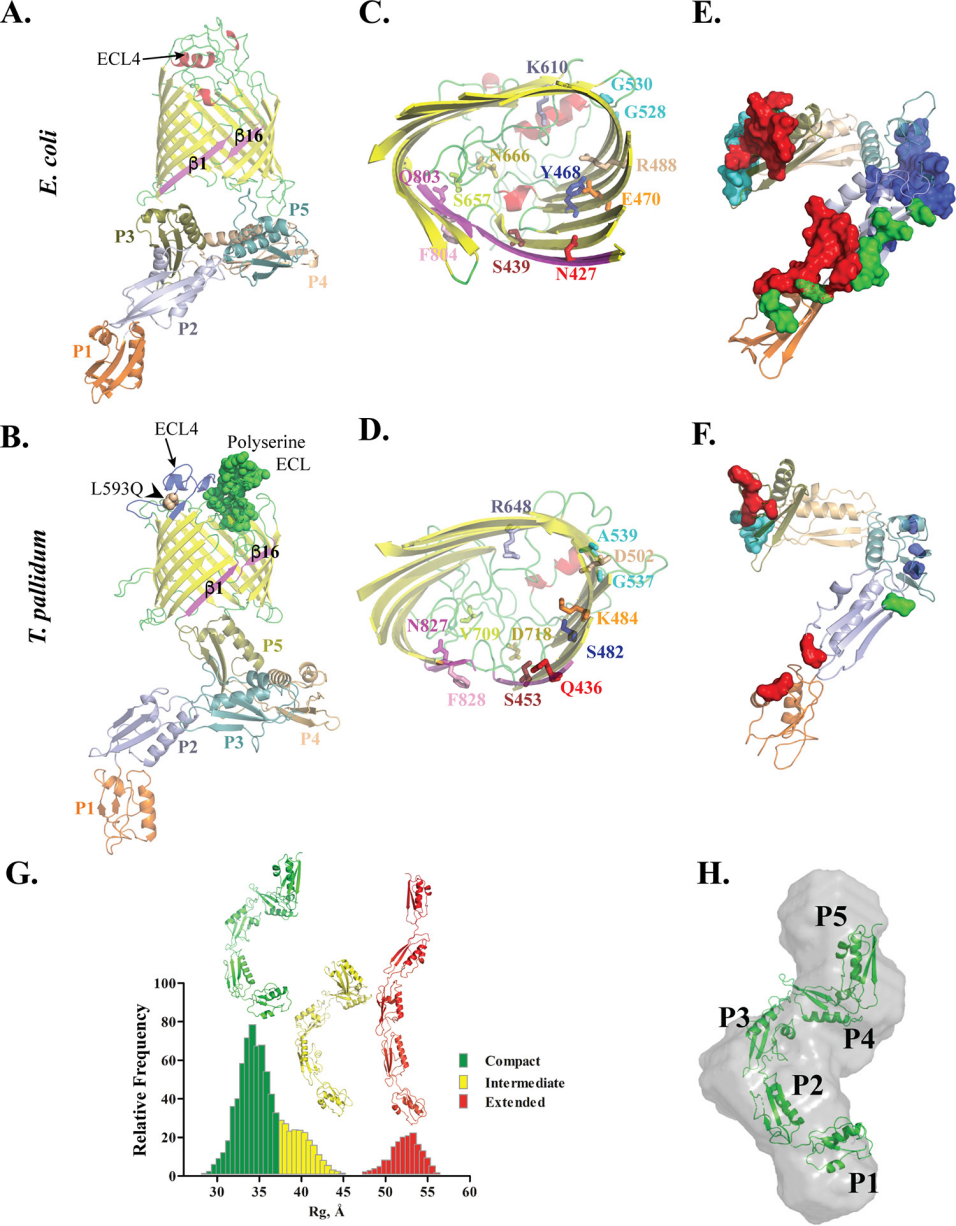
The highly flexible POTRA1-5 of T. pallidum BamA lack BamBCDE-interacting residues. (A and B) Ribbon diagrams for the crystal structure of E. coli BamA (PDB accession number 5D0Q) and the structural model of TP0326. Both proteins are in the same orientation. The arrows indicate ECL4 in both proteins. The β-strands (β1 and β16) forming the lateral gate are shown in magenta. The polyserine tract in ECL7 of TP0326 is displayed as green spheres. A nonconservative amino acid substitution (L593Q) in ECL4 of TP0326 is depicted as an orange sphere with the BCE (shown as a blue ribbon). (C and D) The LptD interaction sites (59) in the β-barrel domain of E. coli BamA (PDB accession number 5D0Q) and their equivalent residues in the 3D model of TP0326 are labeled and shown as sticks. (E and F) BamBCDE-interacting residues of the E. coli POTRA arm (PDB accession number 5D0Q) and their equivalent residues in the 3D model of TP0326 POTRA1-5 are depicted as blue, green, cyan, and red surfaces, respectively. Both POTRA arms (E and F) are in the same orientation. (G) *R_g_* distribution of EOM-generated major conformations for the SAXS data of TP0326 POTRA1-5. Three major conformations (compact, intermediate, and extended) for TP0326 POTRA1-5 are shown in the 3D models. (H) The SAXS envelope (gray surface) of TP0326 POTRA1-5 overlaid with the 3D model of its compact conformation (green 3D model in panel G).

BamA is the central catalyst of the BAM apparatus, the molecular machine that inserts newly synthesized OMPs into the OMs of diderm bacteria (49). In Escherichia coli, BamA consists of a C-terminal β-barrel and an extended N-terminal periplasmic region containing five polypeptide-transport-associated (POTRA) domains (50) (Fig. 1A). Following export across the CM by the Sec translocon, nascent OMPs are transported to the POTRA arm of BamA by the periplasmic chaperones Skp and SurA (51). The POTRA arm then works in concert with the accessory lipoproteins BamBCDE to thread unfolded OMPs into the lumen of the β-barrel (52). To accept its cargo, the BamA β-barrel adopts a conformational state in which the periplasmic aqueous pore is open and the lateral gate is closed (53).

The discovery that T. pallidum contains a BamA ortholog (TP0326), its first characterized rare OMP (54), provided strong support for the assumption that, as in Gram-negative bacteria, the spirochete’s OMPs adopt a β-barrel conformation. Here and elsewhere, we evaluated the integrity of our structural models using MolProbity (55) and WinCoot (56). Our 3D structural model of the BamA β-barrel (Table 1) revealed features typical of Gram-negative BamAs, such as a lateral gate formed by β-strands 1 and 16; a dome comprised of ECL4, -6, and -7 that occludes the barrel’s extracellular opening; and the projection of ECL6 into the barrel interior (44). However, the model also exhibited features suggesting that evolution has tailored T. pallidum’s BamA to catalyze the OM insertion of a distinctive repertoire of OMPs while contending with host-derived immune selection pressures (30, 44). In addition to having a positively charged lumen and different anchoring residues within the barrel for ECL6 (44), TP0326 also harbors an unusual ECL (ECL7) with a two-tiered polyserine-rich tract found only in pathogenic treponemes (44, 57) and a large ECL (ECL4) harboring an antigenically variable BCE (Fig. 1B), which can be targeted by opsonic antibodies (30, 44). Striking molecular differences were also noted between the BAM complex of T. pallidum and its E. coli prototype. The T. pallidum BAM complex is larger than that of E. coli (∼300 to 400 kDa versus 232 kDa), lacks BamBCDE subunits based on genome mining for structural as well as sequence orthologs, and, notably, dissociates in relatively low concentrations of the detergent *n*-dodecyl-β-d-maltoside (DDM) (54, 58).

**TABLE 1:** Structural modeling of the T. pallidum OMPeome

T. pallidum protein	Modeling software^*a*^	Identified structural ortholog; organism; PDB accession no.	% sequence identity with ortholog^*b*^
Components of the BAM complex			
TP0326	ModWeb	BamA; E. coli; 4K3B	19
DUF domain of TP0325	SWISS-MODEL	DUF490 domain of TamB; E. coli; 5VTG	22

Components of the Lpt complex			
TP0515	I-TASSER	LptD; Shigella flexneri; 4Q35	20
TP0784	Phyre2	LptC; Vibrio cholerae; 6MJP	18
TP0785	Phyre2	LptA; E. coli; 2R1A	18
TP0786	Phyre2	LptB; Burkholderia phymatum; 4WBS	50
TP0883	Phyre2	LptF; Vibrio cholerae; 6MJP	15
TP0884	Phyre2	LptG; Vibrio cholerae; 6MJP	17

8-stranded β-barrels			
TP0126	Phyre2, SWISS-MODEL	OmpW; E. coli; 2MHL (NMR structure), 2F1T (crystal structure)	19
TP0733	Phyre2, SWISS-MODEL		23
TP0479	I-TASSER, SWISS-MODEL	OprG; Pseudomonas aeruginosa; 2N6L (NMR structure), 2X27 (crystal structure)	16
TP0698	I-TASSER, SWISS-MODEL		18

FadL-like proteins			
TP0548	trRosetta	Long-chain-fatty-acid transporter FadL; E. coli; 1T1L	17
TP0856	trRosetta		19
TP0858	trRosetta		18
TP0859	trRosetta		17
TP0865	trRosetta		18

Components of efflux systems			
TP0966	trRosetta	OMF (TolC); E. coli; 1TQQ	15
TP0967	trRosetta		16
TP0968	trRosetta		16
TP0969	trRosetta		16
TP0962	Phyre2	IM-spanning domain of MacB; Acinetobacter baumannii; 5GKO	25
TP0963	Phyre2	IM-spanning domain of MacB; Acinetobacter baumannii; 5GKO	27
TP0964	Phyre2	Cytoplasmic domain of MacB; Acinetobacter baumannii; 5GKO	51
TP0965	Phyre2	MacA; E. coli; 5NIK	19
TP0790	Phyre2	AcrB; E. coli; 5V5S	21

T. pallidum repeat proteins: all members of Tprs	trRosetta	No structural template was identified	NA

a ModWeb and SWISS-MODEL, comparative modeling; Phyre2, threading; I-TASSER and trRosetta, *ab initio*. The quality of all models was evaluated with MolProbity (55) and WinCoot (56).

b NA, not applicable.

Recent biochemical and structural studies in E. coli have established that folding of nascent OMPs occurs within the lumen of the BamA β-barrel and, for one substrate, LptD, have identified residues vital to this process (59). To determine whether these essential residues are conserved in T. pallidum BamA, we performed structure-based sequence alignment of the TP0326 3D model with the crystal structures of E. coli BamA (PDB accession numbers 5D0Q and 5D0O) (60). The presence of an LptD ortholog (TP0515 [see below]) in T. pallidum (11) supports the validity of this comparison. Figure 1C and D show that E470, R488, and N666, hydrophilic residues in E. coli BamA essential for LptD folding (59), have been replaced by K484, D502, and D718 in TP0326. In separate study, Hart et al. (61) demonstrated that an E470K mutation in E. coli BamA bypasses the BAM machinery’s requirement for BamBCDE albeit with decreased efficiency. K484, located in the equivalent position in TP0326 (Fig. 1C and D; see also Fig. S1A in the supplemental material), could serve as a comparable “gain-of-function” replacement. While TP0326 maintains a lateral gate for the egress of OMPs, the positions of critical gate residues are swapped in E. coli and T. pallidum (Fig. 1C and D; Fig. S1A): N427 and Q803 on β-strands 1 and 16, respectively, in E. coli BamA (59) have been replaced by Q436 and N827 in TP0326. Thus, whereas the overall mechanism for folding nascent OMPs appears to be preserved in T. pallidum, substitutions in the BamA barrel collectively imply that specific residue interactions differ substantially. Given the much lower replication rate of T. pallidum (30 h *in vivo* versus 20 min for E. coli) (62), these divergences could also serve as an allosteric mechanism to coordinate the kinetics of OMP folding and OM insertion with the growth rate (63). Other factors, such as the difference in lipid compositions between the T. pallidum and Gram-negative OMs (64), particularly the absence of LPS in the former, may also be important in regulating the rate at which the β-barrel of BamA inserts nascent OMPs into the OM bilayer (65).

As shown in Fig. 1E, the contact residues of the five POTRA domains with BamBCDE have been extensively mapped from the crystal structures of the E. coli BAM complex (PDB accession numbers 5D0Q, 5D0O, and 5EKQ) (60, 66). Not surprisingly, most of these interacting residues are absent in the POTRA arm of TP0326 (Fig. 1F). Although T. pallidum contains Skp (TP0327) (54) and a recently identified SurA (TP1016) ortholog (67), the differences in the POTRA arms and the lack of BAM accessory subunits in T. pallidum strongly imply that the syphilis spirochete employs a divergent mechanism for the transfer of nascent OMPs to its BamA β-barrel.

In addition to the BAM complex, E. coli contains a translocation and assembly module (TAM) required for the export of a subset of OMPs, particularly autotransporters, to the cell surface (68, 69). The TAM complex consists of TamA, also a member of the OMP85 superfamily, and a CM protein, TamB, containing a periplasmic DUF490 domain that interacts with the POTRA arm of TamA. *tp0325*, the gene immediately upstream of *tp0326* (Fig. S1B) and likely cotranscribed with it, encodes a large (108-kDa) TamB ortholog with a C-terminal DUF490 domain (68). Webb et al. (70) proposed that T. pallidum harbors a chimeric BAM/TAM OM biogenesis machine. In support of this conjecture, Iqbal and colleagues (71) demonstrated that the TamB ortholog (*bb0794*) of Borrelia burgdorferi, also upstream of *bamA* (*bb0795*), is part of the Lyme disease spirochete’s BAM complex. Homology modeling reveals that, like the TamA interaction partner in E. coli (72), the DUF490 domain of TP0325 is an elongated taco-shaped molecule composed entirely of β-sheets and random coils (Table 1 and Fig. S1C).

Using SAXS, we solved the solution structure of aqueous soluble, monomeric (∼45-kDa), recombinant POTRA1 to -5 (POTRA1-5) of TP0326 (Fig. S2A) to gain insights into the mechanism of transfer of nascent OMPs. The structural model of POTRA1-5 (Fig. 1F) poorly matched the experimental scattering data (χ^2^ = 9.0) (Fig. S2B). Consequently, we assessed whether the SAXS data represent a mixture of conformations by employing an ensemble optimization method (EOM) (73). The EOM generated a three-state ensemble (compact, intermediate, and extended conformations), which dramatically improved the χ^2^ value from 9.0 to 1.0 (Fig. 1G and Fig. S2B). *Ab initio* shape reconstruction from the SAXS data, using 10 independent bead models (see Materials and Methods), yielded a multidomain envelope. The EOM-generated structural model of the compact conformation of POTRA1-5 (Fig. 1G) shows an excellent fit for the *ab initio* molecular envelope (Fig. 1H). Based on the flexibility of the T. pallidum POTRA1-5 solution structure (Fig. 1G and H) and previously solved E. coli BamA structures in multiple conformations (37), we can propose a working model for POTRA-mediated transfer to the BamA channel of nascent treponemal OMPs delivered by the periplasmic chaperones SurA and Skp (Fig. S3). Interaction with the DUF490 domain of TP0325 with TP0326 occurs when the POTRA arm is extended. In this conformation, the periplasmic pore of TP0326 is closed, and the lateral gate is open, permitting the egress of substrates folding within the barrel. The receipt of unfolded, newly exported OMPs from periplasmic chaperones results in the detachment of the POTRA from TP0325, enabling it to assume the bent, “transfer” conformation, which opens the periplasmic pore and closes the lateral gate. This scenario potentially explains the previously observed dissociable nature of the T. pallidum BAM complex (54).

### T. pallidum harbors an LptD ortholog and an Lpt apparatus for transport of an uncharacterized substrate other than LPS.

**FIG 2:**
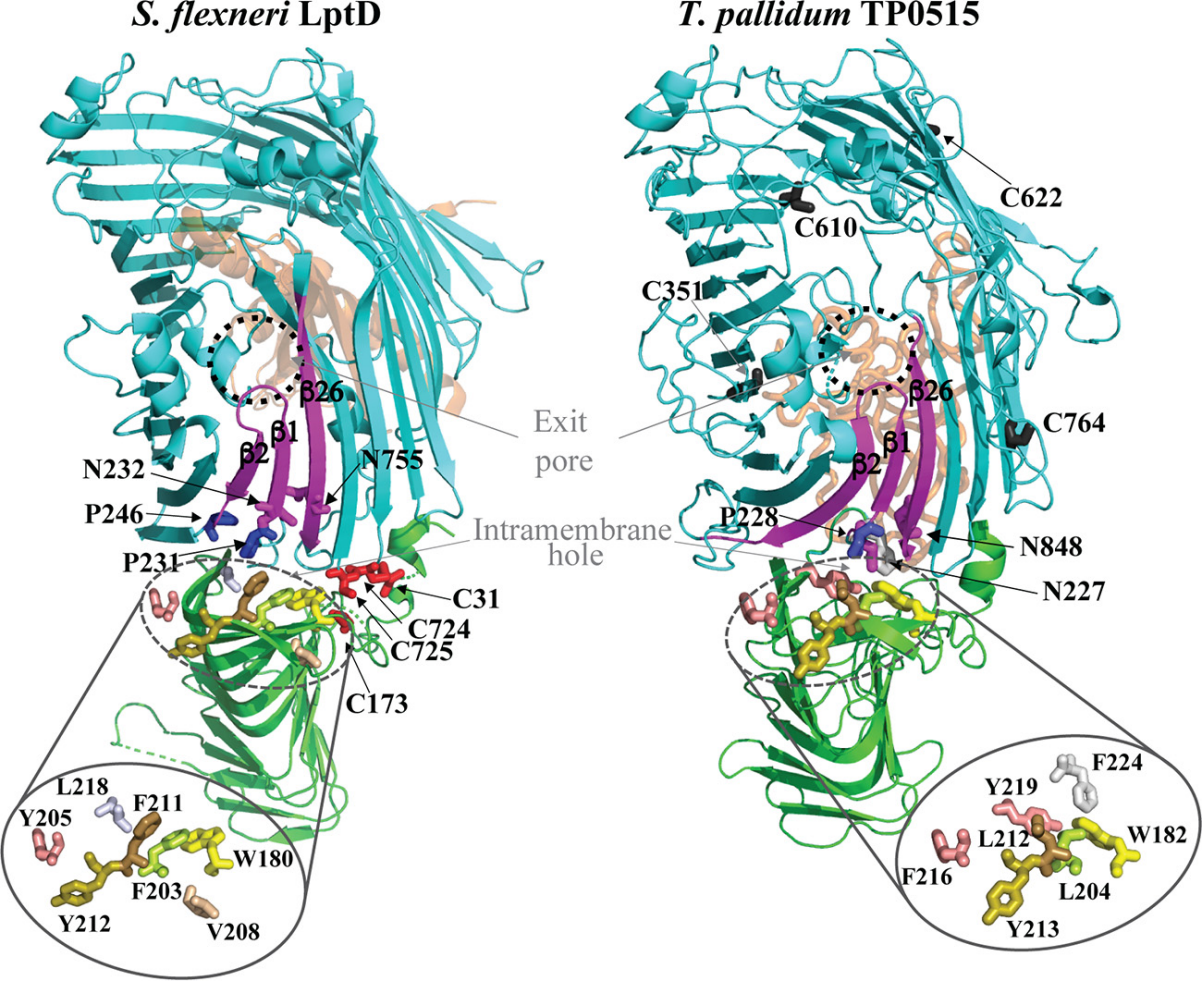
Structural model of T. pallidum LptD (TP0515). Shown are cartoon diagrams of the LptD-LptE complex crystal structure (PDB accession number 4Q35) from Shigella flexneri (left) and the 3D structure model of the T. pallidum LptD ortholog (TP0515) (right). Both proteins are in the same orientation. The β-jelly roll N-terminal domain and 26-stranded β-barrel are shown in green and cyan, respectively. LptE of S. flexneri and the C-terminal extension of TP0515 are depicted as orange ribbons. With both proteins, the β-strands (β1 and β26) of the lateral gate and β2 are shown in magenta. Essential amino acids of the lateral gate (N232, P231, and P246) (38) and the intramembrane hole (W180, F203, Y205, V208, F211, and Y212) (75) and their T. pallidum TP0515 equivalents are displayed as sticks. Residues of intramembrane holes are zoomed-in for clarity. The four Cys residues required for the folding of S. flexneri LptD (76) are shown in red. Cys residues in T. pallidum LptD are shown as dark gray sticks.

The LPS transport (Lpt) machinery consists of seven essential proteins (LptABCDEFG) that mediate the transfer of newly synthesized LPS from the periplasmic side of the CM to the OM (74). The inner membrane (IM) components LptB_2_FG form an ATP binding cassette (ABC) transporter that uses the energy from the hydrolysis of ATP to extract LPS from the CM and push it along the periplasmic jelly roll bridge formed by LptCAD (the so-called “Pez model” for LPS transport) (74). A translocon, consisting of the integral OMP LptD and the LptE lipoprotein “plug” within the lumen of the LptD β-barrel, transfers LPS from the periplasmic bridge to its final destination: the outer leaflet of the OM (74). LptD is a 26-stranded β-barrel with a hydrophilic interior, ECLs that close off the external opening of the barrel and fold into the lumen, a lateral gate between β-strands 1 and 26, and an N-terminal β-jelly roll domain that completes the periplasmic bridge (Fig. 2). A hydrophobic slide within the β-jelly roll domain of LptD guides LPS through a hydrophobic intramembrane hole situated within the bilayer and contiguous with the lateral gate and negatively charged exit pore (Fig. 2) (38, 75). Following transfer from the periplasmic bridge, the lipid A moiety of LPS inserts into the OM through the hydrophobic hole, while the O-antigen is delivered to the extracellular milieu through the lateral gate and exit pore with the assistance of LptE (38, 75). A functional Lpt pathway depends upon the proper folding of LptD, which requires LptE and involves a complex cycle of disulfide bond rearrangements between four conserved cysteines in the β-jelly roll domain and the β-barrel (76).

Because T. pallidum lacks an LPS biosynthetic pathway (6), we were surprised to discover that TP0515, originally identified as a candidate OMP based on secondary structure predictions (43), is a structural ortholog of LptD (11). Moreover, as shown in Fig. S4, with the exception of LptE, T. pallidum contains all the components of the Lpt pathway (Table 1) (74). The 3D model of TP0515, built by an *ab initio* modeling program, I-TASSER (Table 1) (46), contains the hallmark features of Gram-negative LptD described above (Fig. 2). DiscoTope 2.0 analysis of TP0515 predicts that several large ECLs (ECL3, -5, -6, -7, and -12) in its β-barrel domain contain strong BCEs (Fig. S4 and Table S2), which could serve as antigenic targets for host recognition. The likely cargo for the Lpt pathway in T. pallidum is its uncharacterized glycolipids (77), and this probably accounts for structural differences between Gram-negative LptD and TP0515 (Fig. 2). Moreover, it seems reasonable to presume that the putative glycolipid cargo is delivered to the outer leaflet of the OM where it would serve a barrier function. Most notable is an unstructured hydrophilic C-terminal extension in TP0515 that I-TASSER positioned inside the hydrophilic lumen of the β-barrel (Fig. 2); it is tempting to speculate that this region fulfills the functional roles of LptE. The essential hydrophobic residues (75) of the intramembrane hole in TP0515 are well conserved (Fig. 2). Although TP0515 maintains asparagine residues in its lateral gate (N227 and N848 in β1 and β26, respectively), it lacks one of the two essential prolines in the β2 strand (Fig. 2) needed for lateral gate function in Gram-negative LptDs (38), implying somewhat different mechanics of lateral gate function. Whereas Gram-negative LptD has a negatively charged exit pore to expel negatively charged LPS into the outer leaflet of the OM, the exit pore of TP0515 is positively charged (Fig. S5A) and presumably exports an uncharacterized, positively charged glycolipid (77). Interestingly, the exit pore of LptD (LIC11458) from Leptospira interrogans, a spirochete that contains LPS, is negatively charged (Fig. S5A). Finally, TP0515 has four cysteines, but their positions are not conserved with respect to the cysteines in Gram-negative LptD (Fig. 2 and Fig. S5B).

### Eight-stranded β-barrels.

Eight-stranded β-barrels comprise a group of functionally versatile OMPs that are ubiquitous in Gram-negative bacteria (78). Members of this group have either a single-domain β-barrel architecture (e.g., OmpW, Opr, and CarO) (79–81) or a bipartite topology (OmpA) (82) in which the N-terminal domain forms a β-barrel and the periplasmic C-terminal domain interacts with peptidoglycan. Despite their conserved β-barrel architecture, members of this group have low overall sequence similarity, with the ECLs, in particular, showing extensive diversity (83). E. coli OmpW, Pseudomonas aeruginosa OprG, and Acinetobacter baumannii CarO are the most extensively studied representatives of the single-domain subgroup. The crystal structures of OmpW and OprG revealed a narrow hydrophobic channel leading to a lateral opening in the barrel wall (79, 84), while CarO has a hydrophilic channel and lacks a lateral gate (85). It is often stated that OmpW transports small nonpolar substrates (86), OprG transports small neutral amino acids (87), and CarO mediates the uptake of glycine and ornithine (85). However, there is considerable disagreement about the cargos that these proteins handle and their mechanisms of transport (86–88). The dynamic structures of OmpW (PDB accession number 2MHL), OprG (PDB accession number 2N6L), and AlkL of Pseudomonas putida (transports alkanes) (PDB accession number 6QAM) solved by nuclear magnetic resonance (NMR), and their electrostatic potentials, appear to provide a more uniform picture of structure-function relationships in the 8-stranded β-barrels (Fig. S6). The NMR structures revealed that each has a narrow channel, long flexible ECLs, and short β-strands with lengths closely matching the thickness of the OM bilayer (87, 89, 90). These studies, bolstered by molecular simulations, yield a mechanistic model in which substrate transport occurs via dynamic mobility of the ECLs and transient strand separation (89). Eight-stranded β-barrels also contribute to bacterial virulence by virtue of their ECLs (78). As an example, Ail of Yersinia pestis binds to negatively charged extracellular matrix (ECM) components via its positively charged ECLs (83).

**FIG 3:**
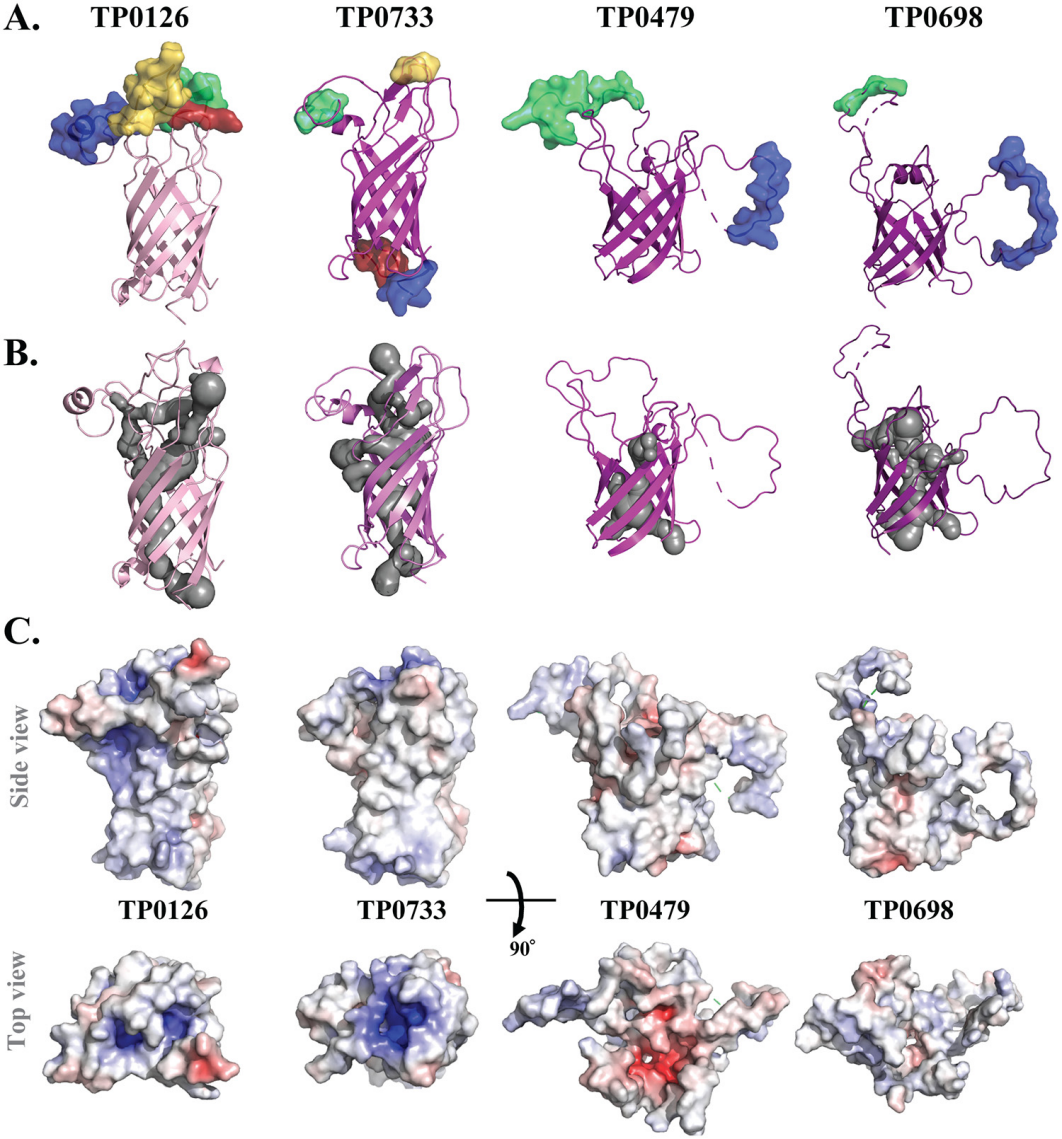
Eight-stranded β-barrels in T. pallidum. Shown are structural models with BCEs (A), predicted luminal cavities (B), and comparative electrostatics (C) for TP0126, TP0733, TP0479, and TP0698. BCEs are shown as transparent surfaces in panel A. The luminal cavities are shown as gray surfaces in panel B.

The T. pallidum Nichols genome encodes two proteins (TP0624 and TP0292) with OmpA-like domains containing peptidoglycan recognition motifs; neither, however, contains a β-barrel domain (91–93). The genome encodes four single-domain 8-stranded β-barrels (TP0126, TP0479, TP0698, and TP0733). TP0126 and TP0733 were identified by *ab initio* modeling using the OmpW crystal structure (PDB accession number 2F1T) (11, 94). TP0479 and TP0698, discovered here from a reanalysis of T. pallidum’s potential β-barrel-forming proteins (43), best fit the crystal structure of OprG (PDB accession number 2X27). Knowing that the NMR and crystal structures for 8-stranded barrels can be at variance, we also used the NMR structures of OmpW (PDB accession number 2MHL) (87) and OprG (PDB accession number 2N6L) (90) as the templates to construct homology models (Table 1). We consider the NMR-based models to be preferable because they provide greater coverage of the TP0126 and TP0733 sequences and yield barrels and ECLs with more open conformations (Fig. 3A). Calculations done using MOLEonline (95) predict that all four proteins have a narrow, continuous channel (Fig. 3B) consistent with possible transport functions. Typical of 8-stranded barrels, structure-based sequence alignments show that the ECLs vary markedly in amino acid composition and length (Fig. S7), with some (e.g., ECL4 of TP0479) likely being highly flexible. As a consequence of this diversity, the ECLs vary in both surface charge (Fig. 3C) and density/distribution of BCEs (Fig. 3A and Table S3). These models support the notion that the syphilis spirochete’s 8-stranded β-barrels transport a variety of small-molecule substrates and engage in diverse interactions at the host-pathogen interface. In other bacteria, 8-stranded β-barrels have shown promise as vaccine candidates (16, 78). The presence of multiple surface-exposed BCEs in the T. pallidum 8-stranded barrels (Fig. 3A) suggests that these proteins could also be targets for a protective immune response. It is noteworthy, therefore, that recombinant TP0126 failed to protect in the rabbit model of experimental syphilis (96). As Haynes et al. noted (96), it remains to be determined whether this negative result reflects the biology of the native protein, which has been proposed to undergo phase variation (94), and/or deficiencies in their immunization/challenge protocol.

### FadL-like 14-stranded β-barrels.

Although biomembranes typically are highly permeable to long-chain fatty acids (LCFAs), Gram-negative OMs are a notable exception because of the permeability barrier created by an outer leaflet composed of LPS (31). Consequently, the transport of LCFAs across the OM of Gram-negative bacteria requires a dedicated transporter (34, 97). Based on the crystal structure of E. coli FadL (PDB accession number 1T1L), the proposed pathway for fatty acid uptake involves two low-affinity binding sites within a hydrophobic groove formed by ECL3 and -4 and a conserved high-affinity site at the entrance of the barrel (Fig. S8A) (34). Once inside the barrel, the substrate disrupts a hydrogen bond between a conserved glycine (G103) in β-strand 3 and a phenylalanine (F3) in a hydrophilic, N-terminal “hatch” region that plugs the lumen (34). Disruption of this bond induces a transient lateral opening at the kink formed by residues SNYG in β-strand 3 (S3 kink); a conserved Asn-Pro-Ala (NPA) motif in the hatch is essential for this substrate-induced conformational change (98). To date, only two other FadL-like OMPs, TbuX of Ralstonia pickettii (PDB accession number 3BRY) and TodX of Pseudomonas putida (PDB accession number 3BRZ), which transport aromatic hydrocarbons, have been structurally characterized (99). Compared to *E. coli* FadL, both structures have similar LCFA high-affinity binding sites but less pronounced S3 kinks and continuous channels extending through their barrels.

Although a previous study proposed that hydrocarbon substrates traverse the channels of TbuX and TodX to reach the periplasm, a recent investigation provided evidence that traversal of the lateral gate would be the preferred mechanism. Although a previous study (99) proposed that hydrocarbon substrates traverse the TbuX and TodX channels to reach the periplasm, a recent investigation (169) provided evidence that aromatic compounds exit their barrels via a lateral gate.

How T. pallidum obtains LCFAs, which it cannot synthesize (7), has been a long-standing question (77). Although the syphilis spirochete’s OM is more permeable to LCFAs than that of E. coli (100), the discovery that it harbors five FadL orthologs (TP0548, TP0856, TP0858, TP0859, and TP0865) (Fig. S8B) (11) argues that the diffusion of LCFAs alone is not sufficient to meet the bacterium’s needs. Moreover, the presence of five paralogous FadL-like OMPs suggests that this family imports a wider range of essential hydrophobic nutrients. Flavins might be one such substrate. In addition to being a fatty acid auxotroph, recent studies by Deka and colleagues (101) have called attention to the spirochete’s “flavin-centric” lifestyle as a basis for many redox biochemical reactions. Flavins have limited water solubility (102), and T. pallidum lacks the ability to synthesis them (6). An alignment percentage matrix (Table S4) shows that T. pallidum FadL-like proteins fall into three subgroups: TP0548 (subgroup I), TP0856 and TP0858 (subgroup II), and TP0859 and TP0865 (subgroup III). To better understand the evolutionary and functional relationships between T. pallidum FadL-like proteins and other bacterial orthologs, we performed a sequence-based phylogenetic analysis of 415 unique FadLs from the Pfam database (http://pfam.xfam.org) (Fig. S9). Interestingly, the T. pallidum FadL-like proteins are much more closely related to R. pickettii TbuX and P. putida TodX than to E. coli FadL and cluster on the same branch of the tree, suggesting evolution from a single precursor.

**FIG 4:**
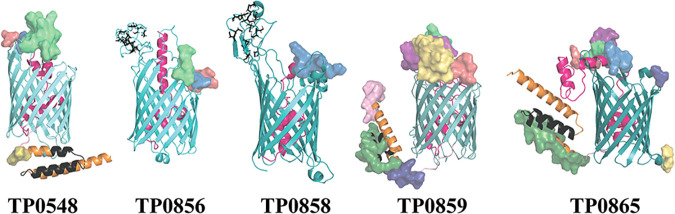
Three T. pallidum FadL-like proteins have a bipartite membrane topology. β-Barrel domains are shown in various cyan shades. In all models, the N-terminal hatch regions are shown in magenta. BCEs are depicted as transparent surfaces. Cysteine residues in the cysteine-rich ECL4 of TP0856 and TP0858 are represented as sticks. The C-terminal helical domains of TP0548, TP0859, and TP0865 are shown in orange and indicated by arrowheads. The TPR motifs in the C-terminal domains are shown in black.

Three-dimensional *ab initio* modeling using either Phyre2 or I-TASSER predicts that all five FadL-like proteins form 14-stranded β-barrels with an unstructured N-terminal hatch within the barrel lumen. However, neither program provided complete coverage for TP0548, TP0859, or TP0865. More specifically, TP0859 and TP0865 have unmodeled stretches at their N and C termini, while TP0548 has an unmodeled stretch at its C terminus (data not shown). Additionally, ECL4 of TP0858 was incomplete. We then used trRosetta (48), an advanced computational program based on direct energy minimization, in an attempt to obtain complete structural models (Fig. 4, Table 1, and Fig. S8B). The complete trRosetta models predict that N- and C-terminal regions of TP0548, TP0859, and TP0865 (subgroups I and III) possess two structural features not seen in the solved crystal structures (34, 99): (i) the N-terminal hatch regions extend from within the barrel to the external surface, where they form α-helices that overlay the entrances (Fig. 4), and (ii) the C-terminal stretches form three α-helical bundle domains attached to the β-barrel by short linkers. A search of all FadL members in the Pfam database failed to identify any other orthologs containing a similar C-terminal domain. Based on its location distal to the β-barrel and predicted surface hydrophilicity, we assigned the C-terminal α-helical bundles to the periplasmic compartment (Fig. 4). MOTIF Search (www.genome.jp/tools/motif) identified a tetratricopeptide repeat (TPR) motif distributed over two antiparallel α-helices of each helical bundle (Fig. 4 and Fig. S8B). Such motifs typically form a hydrophobic groove between the α-helices that mediates protein-protein interactions (103); analysis using YRB (104) revealed a potential hydrophobic cleft in each C-terminal domain (Fig. S10). Phyre2, I-TASSER, and trRosetta all predict that the five FadL orthologs lack the LCFA high-affinity binding site; however, the luminal plug portions of the hatch regions contain either a highly conserved NPA motif (TP0548, TP0856, and TP0858) or an NAA variant (TP0859 and TP0865) (Fig. S8B). Hydrophobicity analysis by the YRB program (104) revealed that each of the treponemal FadL-like proteins contains hydrophobic patches extending from the rim through the barrel (Fig. S11A), providing a potential passageway for hydrophobic substrates into the periplasm. trRosetta also predicts that ECL4 of TP0858 is a long cystine-rich loop comparable to ECL4 of TP0856 (Fig. 4); both could form rigid, disulfide-bonded scaffolds to facilitate the capture of the substrate from the extracellular milieu. In this regard, the extended hydrophobic cleft in ECL4 of TP0858 is particularly noteworthy (Fig. S11B). All five T. pallidum FadL-like proteins harbor one or more predicted conformational BCEs (Fig. 4 and Table S5). Members of subfamilies I and III contain predicted BCEs in their hatch regions, ECLs, and C-terminal extensions, while TP0856 and TP0858 have predicted BCEs only in their ECLs or hatch domains, respectively. These predictions suggest that family members are subject to various degrees of host immunological pressure. Three (*tp0856*, *tp0858*, and *tp0859*) of the five genes are cotranscribed (Fig. S11B and C), suggesting that their gene produces function in a coordinated fashion. It is widely assumed that an effective syphilis vaccine has to target multiple OMPs (105). The coexpression of these three OMPs makes them attractive vaccine candidates since their ECLs would be accessible to antibodies at the same time.

It remains to be determined how *T. pallidum* FadL-like proteins transfer hydrocarbon substrates to the periplasm. Once in the periplasm, however, the transport pathways might diverge based on the physical properties of the substrates, particularly solubility. Conceivably, TP0548, TP0859, and TP0865 transport more hydrophobic substrates. The interaction between their C-terminal α-helical domains and the TRAP transporter (TP0956/TP0957) described by Norgard and colleagues (106, 107) would establish a mechanism to shuttle less-water-soluble nutrients across the periplasm, delivering them to a dedicated CM permease (TP0958). TP0856 and TP0858 would handle more-water-soluble cargo discharged directly into the periplasmic compartment. The periplasmic lipocalin TP0751, which we recently demonstrated has a hydrophobic rim capable of binding small molecules (108), might also serve as a conduit of cargo(s) imported by one or more members of the FadL-like family.

### Treponema pallidum repeat proteins.

Arguably, the most important initial discovery from the T. pallidum Nichols genomic sequence was the Treponema pallidum repeat (Tpr) family of proteins, whose members have sequence homology to the major outer sheath protein (MOSP) of Treponema denticola (7, 109), a known trimeric pore-forming adhesin (110–113) and a major virulence determinant (114). Centurion-Lara and coworkers divided the Tprs into three subfamilies based on sequence relationships (109) (Fig. S12). In the Nichols strain, TprA and TprF are truncated at their C-terminal ends.

Full-length Tprs consist of an extreme amino-terminal stretch (∼50 amino acids [aa]), a conserved N-terminal domain related to the corresponding domain in the N-terminal portion of T. denticola MOSP (MOSP^N^), a central region that varies in both length and sequence among family members (central variable region [CVR]), and a conserved C-terminal domain related to the corresponding domain in MOSP (MOSP^C^) (6, 111). Previous examinations of subfamily I Tprs (TprC/D and TprI) revealed that their MOSP^C^ domains form amphiphilic, trimeric β-barrels with porin activity *in vitro* and are surface exposed in T. pallidum (45, 115). In contrast, the Tpr MOSP^N^ domains and CVRs are water soluble *in vitro* and periplasmic in T. pallidum. The truncated TprF is water soluble *in vitro* and periplasmic in T. pallidum. We undertook further analyses to expand our knowledge of Tpr domain architecture beyond subfamily I to the entire family.

### (i) Domain analysis and modeling predict a tridomain architecture of full-length Tprs.

According to the updated Pfam database (116), all full-length Tprs possess MOSP^N^ and MOSP^C^ domains (Fig. S12). A comparative percentage alignment matrix of all Tpr MOSP^N^ and MOSP^C^ domains revealed less than 23% similarity between the two domains (Table S6). This comparison of amino acid sequences between the two domains suggests that they have distinct evolutionary origins. While the percent similarities within the MOSP^N^ and MOSP^C^ domains are similar (Table S7), their stretches of amino acid similarity differ in distribution (Fig. S13). With both domains, diversity is generated by block insertions as well as amino acid substitutions. Particularly noteworthy in comparisons with the MOSP^N^ and MOSP^C^ domains are the marked differences in the lengths and amino acid compositions of the CVRs (Fig. S12 and S13). Subfamily II CVRs are considerably larger than those in the other subfamilies, also as a result of block insertions, while the CVR of TprK is the smallest (Fig. S13). Collectively, these results imply that the variable length of CVRs, resulting from the wholesale insertion of sequence cassettes, was a major factor in the evolution of the Tprs (111). Previous unsuccessful efforts to predict secondary structures for the CVRs prompted us to consider the possibility that they are unstructured. A search for disorder-based binding regions using the ANCHOR program (117) predicts that several Tprs have short molecular recognition features (MoRFs) (∼6 to 18 aa in length) (Fig. S12). MoRFs are expected to undergo disorder-to-order transitions upon interaction with specific protein partners (118).

**FIG 5:**
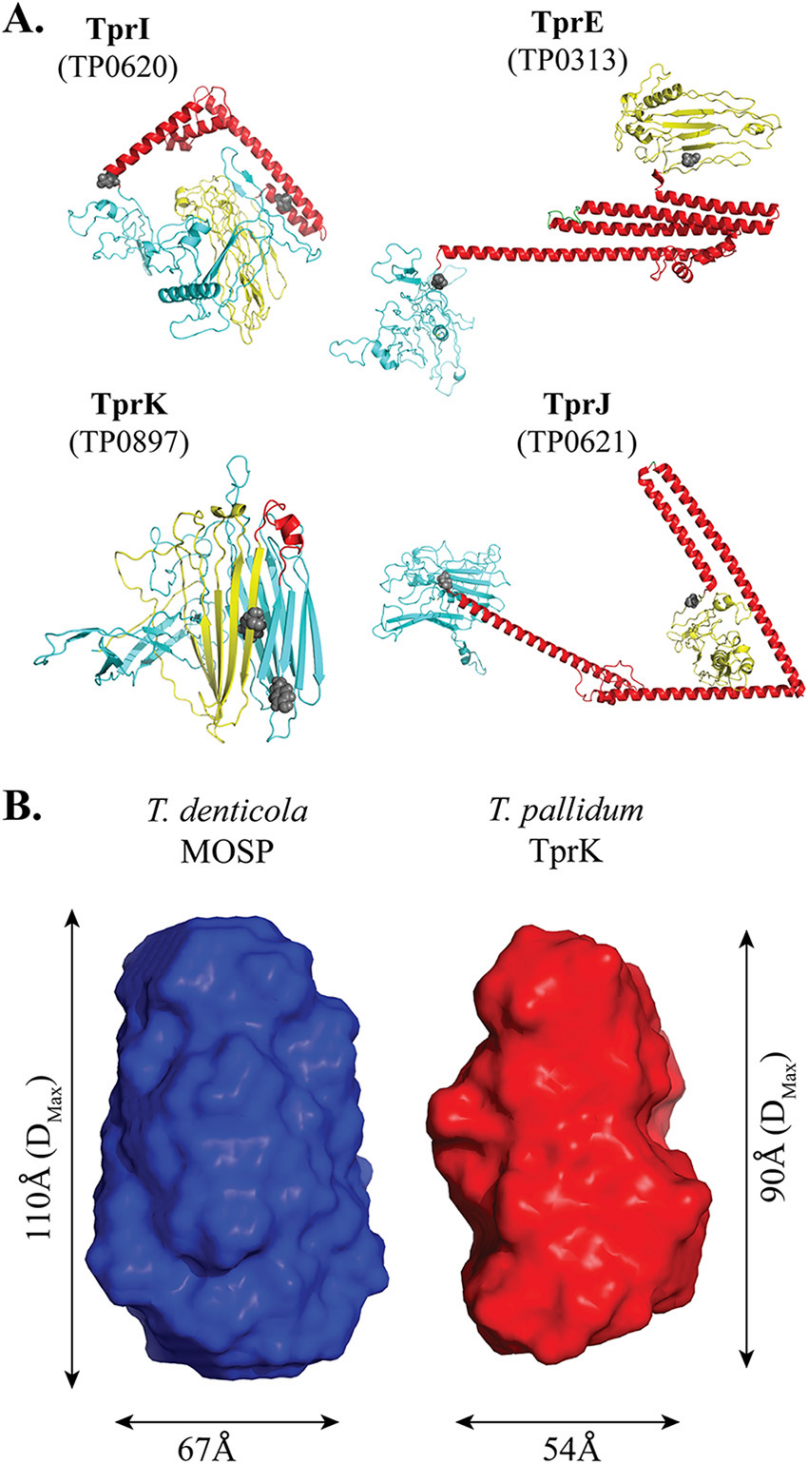
trRosetta modeling of full-length Tprs and solution structures of MOSP^N^ domains of T. denticola MOSP and T. pallidum TprK. (A) MOSP^N^ and MOSP^C^ domains of all Tprs, shown in cyan and yellow, respectively. α-Helices of CVRs are red. Residues corresponding to predicted CVR domain boundaries from Pfam are shown as spheres. (B) *Ab initio* reconstruction of the low-resolution molecular envelopes of T. denticola MOSP and T. pallidum TprK MOSP^N^ domains (blue and red, respectively) calculated using DAMMIN (166) and DAMAVER (167). Both envelopes were generated without enforcing any symmetry. The maximum dimensions (*D*_max_) and the widths of both envelopes are also labeled.

In the absence of sequence or structural orthologs of the Tprs, we previously used the TMBpro Web server to transform the MOSP^C^ domain of TprC/D into a 10-stranded β-barrel topology (30); this conformation is supported by our biophysical data (45). Given that the TMBpro Web server (119) lacks a modeling module to predict secondary and tertiary structures unbiasedly, we used the more accurate *ab initio* modeling program trRosetta for the full-length Tprs (Table 1). trRosetta was unable to generate a β-barrel model for either the full-length polypeptides or individual domains, presumably reflecting the lack of structural templates in the databases. However, it predicted that all nine full-length Tprs have a tridomain architecture with domain boundaries (Fig. 5A) corresponding closely to those predicted by Pfam (Fig. S12). The MOSP^N^ and MOSP^C^ domains are predominantly β-stranded, in agreement with secondary structure analyses previously obtained by circular dichroism (CD) spectroscopy (45, 115). Importantly, trRosetta predicts that all CVRs, including the diminutive CVR of TprK, consist exclusively of α-helices (Fig. 5A). The predicted conservation of secondary structure for the CVRs is noteworthy given their low degree of sequence similarity (Fig. S13 and Table S7).

### (ii) SAXS supports the bipartite membrane topology of all Tprs.

Taken together, the above-described findings indicate that full-length Tprs have a tridomain architecture and a bipartite membrane topology. We used SAXS to garner additional experimental evidence for the bipartite membrane topology of Tprs. For these investigations, we chose MOSP^N^ of T. denticola MOSP as a structural surrogate for the corresponding Tpr domains and MOSP^N^ of TprK given the widespread belief that all seven of its variable regions occur within a β-barrel formed by the full-length polypeptide (120–122). The MOSP^N^ domains of both proteins could be purified by Ni-nitrilotriacetic acid (NTA) chromatography without denaturants or detergents and, by size exclusion chromatography (SEC), eluted as monomers with molecular weights of ∼25 kDa (Fig. S14A). In SAXS experiments, the scattering curves and intraparticle distance distribution functions [*P*(*r*)] of both domains showed correct folding without aggregation (Fig. S14B and C). As with TprF (45), *ab initio* envelope reconstructions from the SAXS data reveal rigid, nonglobular shapes (Fig. 5B).

### T. pallidum efflux systems and outer membrane factors.

Bacteria not only need to import nutrients but also need to expel toxigenic molecules of endogenous or exogenous origin (123). The capacity of numerous bacterial species to tolerate antibiotics and other toxic compounds elaborated by the commensal flora and/or host defenses arises in large part from the activity of efflux systems (124). Additionally, efflux systems enhance the virulence potential of bacterial pathogens by protecting against reactive oxygen species (125).

In Gram-negative bacteria, multicomponent efflux systems span both the inner and outer membranes and consist of an OMF, an inner membrane-spanning transporter (pump), and a periplasmic adaptor that connects the two (41). The pumps for these multicomponent systems fall into two families: ATP binding cassette (ABC) and resistance-nodulation-cell division (RND) transporters. ABC transporters (e.g., E. coli MacB) consist of an N-terminal nucleotide binding domain (NBD) and four transmembrane (TM) helices and hydrolyze ATP to drive transport (126). RND transporters (e.g., E. coli AcrB) consist of a TM domain with 12 α-helices within the CM, a porter domain in the periplasm containing the substrate binding pocket, and a docking domain that connects the pump to the adaptor; RND transporters are powered by the proton motive force (126). In both systems, OMF monomers (e.g., E. coli TolC) form a trimer with 3-fold symmetry in which 4 β-strands and 6 α-helices from each subunit associate to form a 12-stranded β-barrel within the OM and an α-helical barrel within the periplasm, which interacts with a hexameric adaptor (e.g., AcrA and MacA) (127, 128). The periplasmic adaptor and the IM transporter maintain a stable association, while the OMF interacts transiently with the periplasmic adaptor. As a result, in E. coli, TolC can associate with adaptors for both pumps (129).

**FIG 6:**
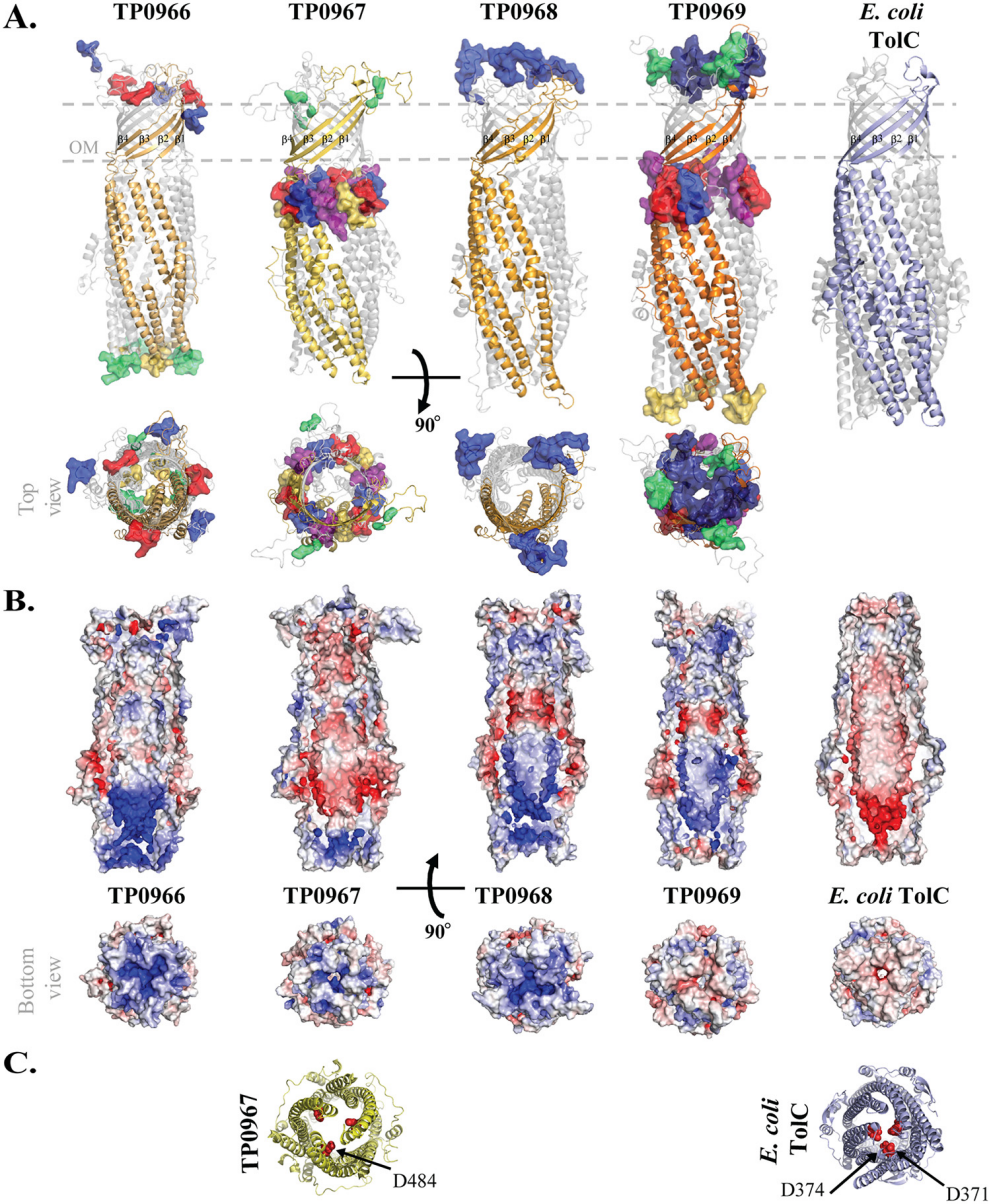
Channels of T. pallidum OMFs have dissimilar electrostatic potentials. (A) Ribbon diagrams for trimeric structural models of T. pallidum OMFs and the crystal structure of E. coli TolC (PDB accession number 1TQQ). One protomer in all four OMFs is shown in various shades of gold, while the TolC protomer is blue. BCEs are displayed as surfaces. All proteins are in the same orientation, with β-strands labeled. (B) Comparative electrostatics (same orientation) of T. pallidum OMFs and TolC. (C) View from the periplasmic entrance showing the aspartate ring (red spheres) of TP0967 and E. coli TolC. Both proteins are in the same orientation.

An investigation by Brautigam and colleagues of Tp34 (TP0971), a T. pallidum lipoprotein involved in metal homeostasis, resulted in the serendipitous discovery of a large operon (*tp0959* to *tp0972*) encoding a MacB transporter (TP0962, TP0963, and TP0964), a periplasmic adaptor (TP0965), and four OMFs (TP0966 to TP0969) (130). In contrast to conventional MacB (128, 131), which is a homodimer, the MacB transporter in T. pallidum consists of a heterodimeric TM component (TP0962/TP0963) and two identical NBDs (TP0964) (130). We identified an ortholog (TP0790) of the RND transporter AcrB elsewhere in the T. pallidum genome (11). Multiple sequence alignment (MSA) (Table S8) and phylogenetic analysis (Fig. S15A) revealed that the four T. pallidum OMFs share 30% to 40% sequence similarity and cluster on a single branch close to E. coli TolC and Salmonella enterica serovar Typhi St50. 3D models of the TP0966, TP0967, TP0968, and TP0969 protomers (Fig. 6A, Table 1, and Fig. S15B) have a typical TolC-like topology consisting of four β-strands with two large ECLs and six α-helices. Threefold symmetry was applied to assemble trimers for the four OMFs. Whereas the predicted topologies of the T. pallidum OMFs closely overlap that of TolC (PDB accession number 1TQQ) (132) (Fig. 6A and Table S9), the electrostatics of the channels differ greatly from that of TolC as well as each other. Notably, the TolC channel is entirely negative, while the T. pallidum OMF channels contain variable patches of basic and acidic residues (Fig. 6B). Interestingly, the TP0967 channel, the most negatively charged of the four, also contains an aspartate ring (Fig. 6C), which, in TolC, determines ion selectivity (133). As with the genes encoding the FadL-like proteins, the cotranscription of OMF genes (*tp0966* to *tp0969*) implies that they function in parallel (130). Also, as with the *fadL*-like genes, cotranscription makes them attractive vaccine candidates. DiscoTope 2.0 predicts that all four trimers, most notably TP0969, contain surface-exposed BCEs (Fig. 6A and Table S10).

**FIG 7:**
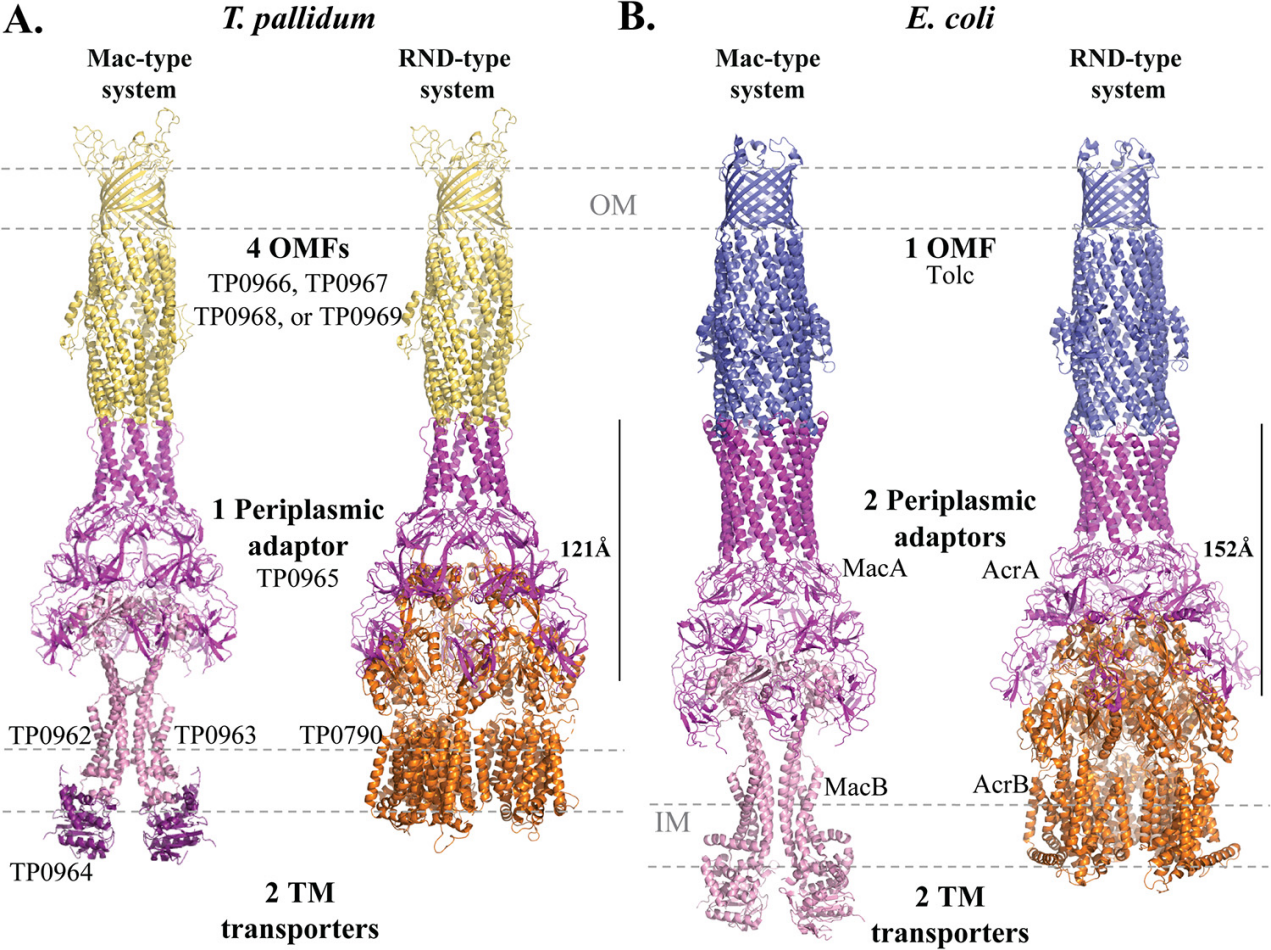
Modeling of T. pallidum periplasmic adaptors and TM transporters. (A) Structural models of the T. pallidum periplasmic adaptor (TP0965), the Mac-like TM transporter (TP0962/TP0963/TP964), and the RND-like TM transporter (TP0790) and OMF (TP0969). (B) Cryo-EM structures of E. coli Mac-type (PDB accession number 5NIK) and Acr-type (PDB accession number 5V5S) efflux systems.

We used Phyre2 and PyMOL to construct a hexameric assembly of the periplasmic adaptor (TP0965), which exhibits a typical MacA-like funnel but is shorter than E. coli MacA (∼121 Å versus ∼152 Å, respectively) (Fig. 7A and B). Because our mining of the T. pallidum genome revealed only one adaptor, we hypothesize that it functions in both efflux systems. This conjecture is based on the mixed electrostatic charges at the interface of TP0965 with the two efflux pumps (TP0962/TP0963/TP0964 and TP0790) (Fig. S16A). In E. coli, the positively charged periplasmic interface of MacB interacts with the negatively charged projections of MacA, while the converse is true for AcrB and AcrA (Fig. S16B). We next assembled the complete Mac and Acr efflux system in T. pallidum (Fig. 7B) using the cryo-electron microscopy (cryo-EM) structures of E. coli Mac and Acr (PDB accession numbers 5NIK and 5V5S, respectively) (127, 128) (Fig. S16B).

In contrast to E. coli, in which one OMF (TolC) interacts with multiple adaptors (129), T. pallidum appears to have taken a different interactome approach to achieve substrate versatility of its efflux systems. The spirochete’s use of four OMFs, one periplasmic adaptor, and two TM exporters theoretically enables it to contend with a wide range of biochemical threats encountered in different tissues during the protracted course of this chronic disease. Because of its extraordinary invasiveness, T. pallidum is usually conceived of as a “loner” pathogen (6). However, during sexual activity, syphilis spirochetes are often inoculated into sites that teem with commensal microorganisms (4). The efflux pumps are likely a critical component of the spirochete’s early survival strategy, particularly in sites such as the female genital tract and the rectal mucosa, which have complex microbial communities that produce a variety of bactericidal small molecules and peptides. In addition, one can assume that mucosal epithelial cells respond to the invader by secreting antimicrobial peptides (AMPs) and sequestering transition metals (“nutritional immunity”) (134). A mouse model of lower female genital tract infection with Neisseria gonorrhoeae provides precedent for this notion; the absence of the MtrC-MtrD-MtrE efflux system decreases the survival of gonococci (135). Given the complex cellular inflammatory response that T. pallidum elicits in all stages of syphilis, the spirochete’s need to deal with toxigenic and redox-active molecules of exogenous origins likely extends beyond early infection (6). T. pallidum’s ability to evade host defenses and establish persistence due to its OM molecular architecture is the crux of its designation as the stealth pathogen (6). The potential ability to extrude different combinations of noxious molecules in diverse milieus, supported by its OMF repertoire, likely adds a new structure-function dimension to the concept of stealth pathogenicity.

### Concluding remarks.

The OM is a durable, adaptable permeability barrier that enables Gram-negative bacteria to thrive in unpredictable, and often hostile, environments as free-living organisms, pathogens, or both (31). After more than 50 years of intensive investigation, the ultrastructure, constituents, and biogenesis of the Gram-negative OM are reasonably well understood (23, 52). The T. pallidum OM must also support homeostatic, protective, and virulence-related functions but for a bacterium with a markedly limited biosynthetic capacity and an exclusively pathogenic, invasive lifestyle capable of causing protracted, and even lifelong, infection in humans (2, 136). We have known for years that the properties, composition, and molecular architecture of the T. pallidum OM differ greatly from those of its Gram-negative counterparts (6, 11, 137). Cryo-EM has revealed that spatial relationships between the OM of T. pallidum and the other compartments of the cell envelope differ greatly from those of Gram-negative bacteria (9, 10). Previously, we identified candidate OMPs in the spirochete based on their predicted ability to adopt a β-barrel conformation (43). The characterization of these molecules and their interaction partners using structure-based approaches (30, 44) represents an important step toward deconvoluting the spirochete’s enigmatic mechanisms for persistence and devising strategies to elicit protective immunity.

How T. pallidum obtains the vast array of nutrients required to sustain infection of its obligate human host is a long-standing conundrum of syphilis pathogenesis, well predating the sequencing of the spirochete’s minimalist genome (7, 8, 138, 139). The analyses presented here reveal that much of the answer to the enigma resides in the previously unsuspected diversity and complexity of its OMPeome. T. pallidum differs from many (although not all) Gram-negative bacteria in that it contains multiple paralogous OMP families. We postulate that each family enables the bacterium to import a different “class” of substrate and that members within a family have different substrate specificities within that class. With the important exception of the Tprs, T. pallidum has achieved this functional diversity by utilizing known structural orthologs as evolutionary templates. The multidomain Tprs, on the other hand, which have no Gram-negative counterpart, appear to have evolved under a combination of pressures related to the spirochete’s architectural plan (i.e., the need to stabilize the OM by tethering it to the peptidoglycan sacculus [9, 10]) in addition to its metabolic requirements (i.e., the need for porin-like proteins to passively import numerous small molecules). While the bacterium requires an OMP repertoire with broad importation capabilities, it does not need high copy numbers of individual importers given its dramatically slower replication time (∼30 h) than that of Gram-negative bacteria (62, 140, 141). From the standpoint of immune evasion, the benefits of this evolutionary path seem readily apparent. T. pallidum appears to contend with a less obvious adverse consequence of this broad importation strategy, the incidental uptake of cytotoxic small molecules, by deploying a robust combinatorial efflux system to function in concert with its importers.

As an obligate human pathogen with no known environmental or zoonotic reservoir that has remained exquisitely sensitive to penicillin G for the past 75 years (2, 142, 143), one would expect that eradication of T. pallidum could be accomplished by aggressive public health measures. The explosive increase in new syphilis cases in the United States and globally during the past 20 years clearly argues otherwise (1, 4, 144). Consequently, there is a growing sense of urgency about the need for a vaccine as a cornerstone of a global containment strategy (105, 145). Because T. pallidum is an extracellular bacterium (6, 8), an effective vaccine must elicit antibodies against antigens on the bacterial surface (105, 145). Years of investigation of the molecular architecture of the T. pallidum outer membrane have shown that the principal candidates for syphilis vaccine design are contained within the spirochete’s OMPeome (6, 11, 108, 137). The structural models described here provide investigators, for the first time, with a platform for a rational, systematic investigation of the combination of OMPs needed to elicit long-lived protection. With β-barrel proteins, protective antibodies must be directed against BCEs located in ECLs. Applying the principles of structural vaccinology will enable investigators to ascertain that antibodies elicited in syphilis vaccine trials with animal models precisely target the Achilles’ heels of candidate OMP vaccinogens: their ECLs.

## MATERIALS AND METHODS

### Ethics statement.

All animal experimentation was conducted according to the *Guide for the Care and Use of Laboratory Animals*, 8th ed. (146), and in accordance with protocols reviewed and approved by the UConn Health Institutional Animal Care and Use Committee under the auspices of Animal Welfare Assurance A3471-01.

### Propagation of T. pallidum.

T. pallidum (Nichols strain) was propagated by intratesticular inoculation of adult male New Zealand White rabbits and harvested in CMRL medium (Gibco) supplemented with 20% heat-inactivated normal rabbit serum at peak orchitis as previously described (54). Spirochetes were enumerated by dark-field microscopy using a Petroff-Hausser counting chamber (Hausser Scientific).

### Cloning procedures.

Recombinant constructs and oligonucleotide primers used in this study are listed in Table S10 in the supplemental material. All cloned constructs are based on the T. pallidum Nichols strain. Constructs for POTRA1-5 of BamA (TP0326) and the MOSP^N^ domain of T. denticola strain ATCC 35405 without its signal sequence were described previously (44, 67). DNA encoding the MOSP^N^ domain (amino acid residues 29 through 259) of TprK (TP0897) (GenBank accession number WP_010882340) without its signal sequence was PCR amplified from the pUK57 plasmid harboring an E. coli codon-optimized version of *tprK* (synthesized by GenScript). The resulting amplicon was cloned into linearized pET28a in frame with the N-terminal His_6_ tag using in-fusion cloning (147) (TaKaRa).

### Expression and purification.

All constructs in this study were overexpressed in E. coli OverExpress C41(DE3) (Lucigen) at 20°C. Expression was induced at an optical density at 600 nm (OD_600_) of ∼0.4 by the addition of isopropyl-β-d-1-thiogalactopyranoside (IPTG) to a final concentration of 0.5 mM, followed by 12 to 44 h of growth. Following induction, cells were harvested at 7,000 × *g* for 20 min at 4°C. The pellet was lysed in Tris-NaCl buffer (20 mM Tris-HCl [pH 7.5], 150 mM NaCl) supplemented with a protease inhibitor cocktail (PIC; Sigma-Aldrich). The lysate was centrifuged at 14,000 rpm for 40 min at 4°C to remove insoluble proteins and cell debris. The supernatant was mixed with Ni-NTA resin (Qiagen, USA) for 1 h and washed with Tris-NaCl buffer, followed by another wash with the same buffer containing 40 mM imidazole. Elution was carried out in Tris-NaCl buffer with 400 mM imidazole at pH 7.5. Elution fractions were pooled and subjected to size exclusion chromatography (SEC) using a Superdex-200 HR 16/60 column (GE Healthcare Life Sciences) preequilibrated with a solution containing 50 mM Tris (pH 7.5), 100 mM NaCl, and 1 mM dithiothreitol (DTT). The purity of all proteins was verified by sodium dodecyl sulfate-polyacrylamide gel electrophoresis (SDS-PAGE). The final proteins (POTRA arm and the MOSP^N^ domains of TprK and MOSP) were concentrated to 1 to 3 mg/ml using an Amicon filtration device, flash-frozen in liquid nitrogen, and stored at −80°C until use.

### 3D modeling of T. pallidum OMPs.

To predict the 3D structures of recently identified T. pallidum OMPs (11), amino acid sequences were submitted to the Phyre2 server (http://www.sbg.bio.ic.ac.uk/phyre2/html/page.cgi?id=index) (47). A confidence score of 90% or higher (47) between the template model and the target match was set as a cutoff value (47). The high confidence level (90%) indicates that the query protein is likely to adopt the overall predicted β-barrel at high accuracy (47). Proteins that did not meet the 90% threshold or had unmodeled regions in Phyre2 were submitted to I-TASSER and/or trRosetta (46). The accuracy of 3D models from I-TASSER was quantitatively measured by the *C* score (148). The structural model of BamA was made, as described previously by Luthra et al. (44), using the solved structure of full-length BamA (PDB accession number 3KGP) from N. gonorrhoeae (149). The quality of all models was evaluated with MolProbity (55). All Tpr models were made using trRosetta (46). Steric clashes in each model were evaluated using WinCoot (56). Structural modeling and sequence identities are summarized in Table 1.

### 3D modeling data availability.

Atomic coordinates for all structural models of the T. pallidum OMPeome are downloadable from https://drive.google.com/file/d/1EurEnlwAiqtsUm8t-jC3Xuz5e7nV45mT/view?usp=sharing&export=download.

### Modeling and assembly of the T. pallidum Lpt complex.

The Phyre2 server (47) was used to build the initial 3D structural model of T. pallidum LptB (TP0786), LptF (TP0883), LptG (TP0884), LptC (TP0784), and LptA (TP0785) orthologs. Next, the crystal structure of Vibrio cholerae LptB_2_FGC (PDB accession number 6MJP) (150) was applied as a template to assemble a structural model for T. pallidum LptB_2_FGC (TP0786_2_-TP0883-TP0884-TP0784). Finally, T. pallidum LptA (TP0785) was manually aligned between the N-terminal domain of LptD (TP0515) and LptC (TP0784).

### Modeling and assembly of T. pallidum efflux systems.

The crystal structure of trimeric E. coli TolC (PDB accession number 1TQQ) (132) was used to build trimers of T. pallidum OMFs TP0966, TP0967, TP0968, and TP0969 using WinCoot (56). Structural models of TP0790, TP0962/TP0963, TP0964, and TP0965 were made in Phyre2 (47). The cryo-EM structures of the MacAB-TolC (PDB accession number 5NIK) (128) and AcrAB-TolC (PDB accession number 5V5S) (127) efflux pump-OMF complexes were used as the templates to assemble T. pallidum ABC-type and RND-based pumps with the periplasmic adaptor (TP0965) and OMFs (TP0966, TP0967, TP0968, and TP0969).

### Bioinformatics.

DOG 2.0 (151) was used to delineate domains and extracellular loops of T. pallidum OMPs on the primary (one-dimensional [1D]) protein structures. Structures were aligned using the secondary structure matching (SSM) superimposition algorithm in WinCoot (56). All structural figures were rendered in PyMOL (https://pymol.org/) (152). MSA was performed using ClustalW with the GONNET matrix in MacVector. Sequence identity matrices were calculated in Clustal Omega using the default parameters (153). PROMALS3D (154) and ESPript3.0 (155) were used to generate structure-based sequence alignments. The electrostatic potential of 3D structures was calculated using the adaptive Poisson-Boltzmann solver (APBS) (156). Discontinuous BCEs were predicted from each 3D model using a threshold of −3.7 with the DiscoTope 2.0 server (157). The score for BCEs is calculated as a combination of propensity scores of amino acids in spatial proximity and contact numbers. The residue contact number is the number of C_α_ atoms in the antigen within a distance of 10 Å (158). All phylogenetic analyses were performed using Clustal Omega (153) and visualized using Interactive Tree of Life (iTOL) (V4) (159).

### Acquisition of small-angle X-ray scattering data.

Online SEC–small-angle X-ray scattering (SAXS) at the 16 LiX beamline (160) of National Synchrotron Light Source II (Brookhaven National Laboratory) was used to collect scattering data for the MOSP^N^ domain of TprK. One hundred fifty microliters (∼200 μM) of a protein sample (TprK) was injected into a Superdex Increase 200 10/300 GL column (GE Healthcare) at a flow rate of 0.45 ml/min. Scattering images were collected continuously at 1-s exposures per frame. The data processing program pyXS (161) was used for scaling, integration, and averaging of individual scattering images after inspection for aggregation. SAXS data of MOSP^N^ of T. denticola MOSP were also collected at the 16 LiX beamline (160) under static data collection conditions using a 1.0-mg/ml protein concentration. pyXS (161) was used to determine the scattering contribution of the MOSP^N^ domain of T. denticola MOSP by subtracting the background scattering of the buffer.

The SAXS data of TP0326 POTRA1-5 were collected at the Cornell High-Energy Synchrotron Source (CHESS) beamline using a dual Pilatus 100 K-S SAXS/wide-angle X-ray scattering (WAXS) detector, and background subtraction of SAXS buffer was performed using the open-source software RAW (162).

### SAXS analysis and model building.

The ATSAS software package (163) was used to generate 3D *ab initio* envelopes of BamA POTRA1-5, T. denticola MOSP^N^, and TprK MOSP^N^ from one-dimensional SAXS profiles. Radii of gyration (*R_g_*), deduced from the Guinier region of the Guinier plots, were computed using PRIMUS (164). GNOM (165) was used to generate a well-behaved *P*(*r*) curve. *Ab initio* envelope reconstructions were performed using DAMMIN (166) and DAMAVER (167). Theoretical scattering curves were compared against experimental scattering data using FoXS (168). The predicted flexibility of POTRA1-5 was determined using the ensemble optimization method (EOM) (73).

### Operon prediction of FadL-like genes in T. pallidum.

The FGENESB server (http://www.softberry.com/berry.phtml?topic=fgenesb&group=programs&subgroup=gfindb) was used for operon prediction for the region encompassing nucleotide positions 937198 to 932966 of the T. pallidum Nichols strain (NCBI accession number NC_021490.2) genome carrying *tp0856*, *tp0858*, and *tp0859*, including intergenic regions between *tp0856* and *tp0858* and between *tp0858* and *tp0859*.

### Expression analysis of *fadL*-like genes.

RNA was extracted from testicular tissue of four rabbits inoculated with the T. pallidum Nichols strain as previously described (54). The RNA concentration was measured using a Nanodrop spectrophotometer (Thermo Scientific). cDNA synthesis was performed using the high-capacity cDNA archive kit (Applied Biosystems) according to the manufacturer’s instructions. Primers used to amply transcripts for *tp0856*, *tp0858*, *tp0859*, and the intergenic regions between *tp0856*, *tp0858*, and *tp0859* are listed in Table S10. Amplification reactions were performed in a GeneAmp 9700 PCR system (Applied Biosystems) as follows: 1 cycle at 98°C for 3 min, followed by 35 cycles of 98°C for 10 s, 55°C for 15 s, and 72°C for 10 s and 1 cycle of 72°C for 3 min.
