# Supplementary material for: Structural Modeling of the Treponema pallidum Outer Membrane Protein Repertoire: a Road Map for Deconvolution of Syphilis Pathogenesis and Development of a Syphilis Vaccine
Source: J Bacteriol. 2021 Jul 8;203(15):e00082-21. doi: 10.1128/JB.00082-21 (PMC8407342; doi:10.1128/JB.00082-21)
Supplement: Supplemental file 1 — Fig. S1 to S16 and Tables S1 to S10. Download JB00082-21-s0001.pdf, PDF file, 1.39 MB [file JB00082-21-s0001.pdf]

Structural modeling of the *Treponema pallidum* OMPeome: a roadmap for deconvolution of syphilis pathogenesis and development of a syphilis vaccine

Supplemental material

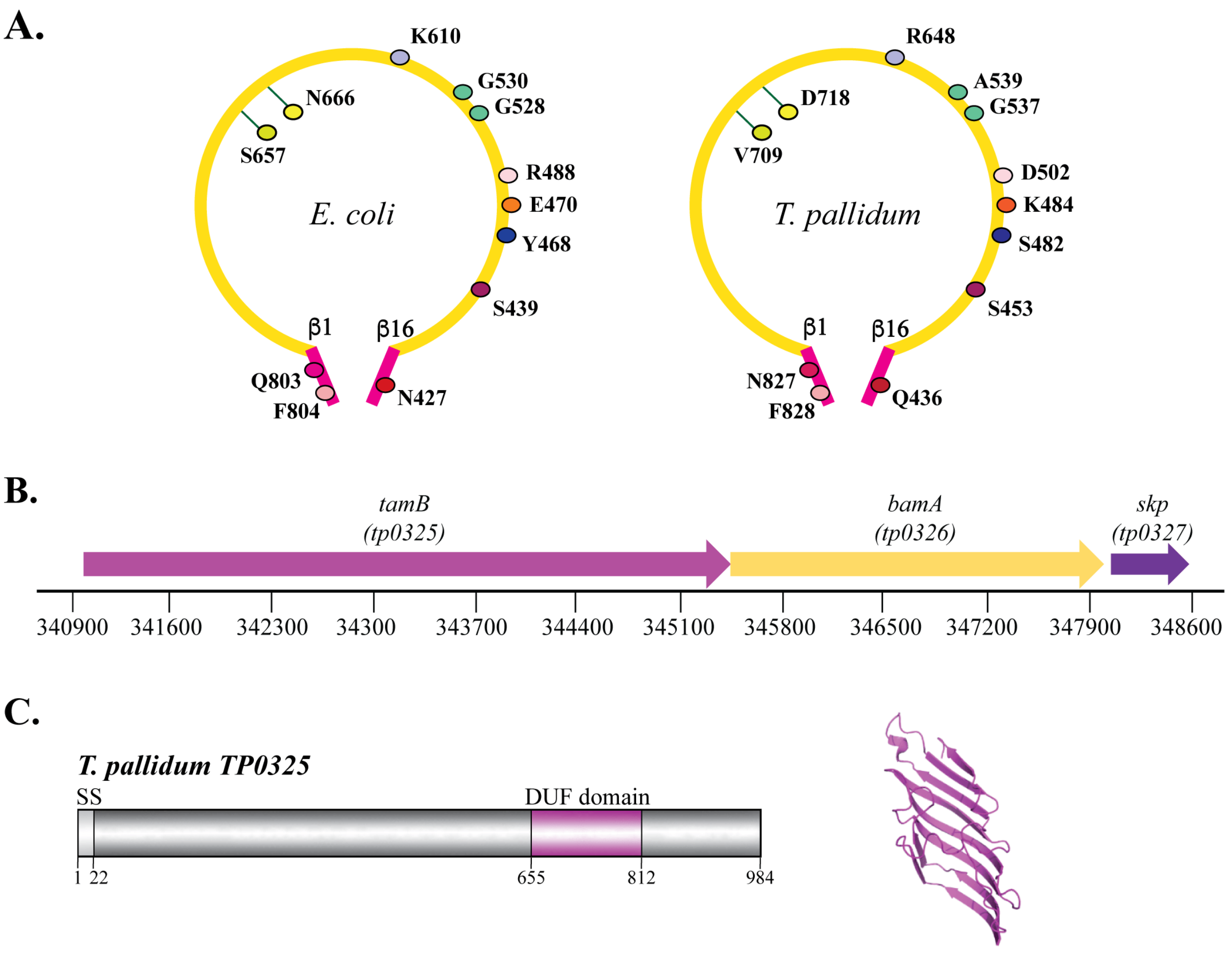

Fig. S1. A. Cartoon schematic of the LptD interaction sites in *E.coli* BamA (PDB ID: 5D0Q) (left panel) and equivalent residues in the β-barrel of TP0326 (right panel). Yellow circled residues with green sticks are located in extracellular loops, while the remaining circled residues are present in β-strands. The lateral gates are colored in magenta. **B.** Schematic depiction of the *tp0325*(*tamB*)-*tp0326*(*bamA*)-*tp0327*(*skp*) operon. **C.** Schematic representation of TP0325 (left) and the 3D structural model of its DUF490 domain (right).

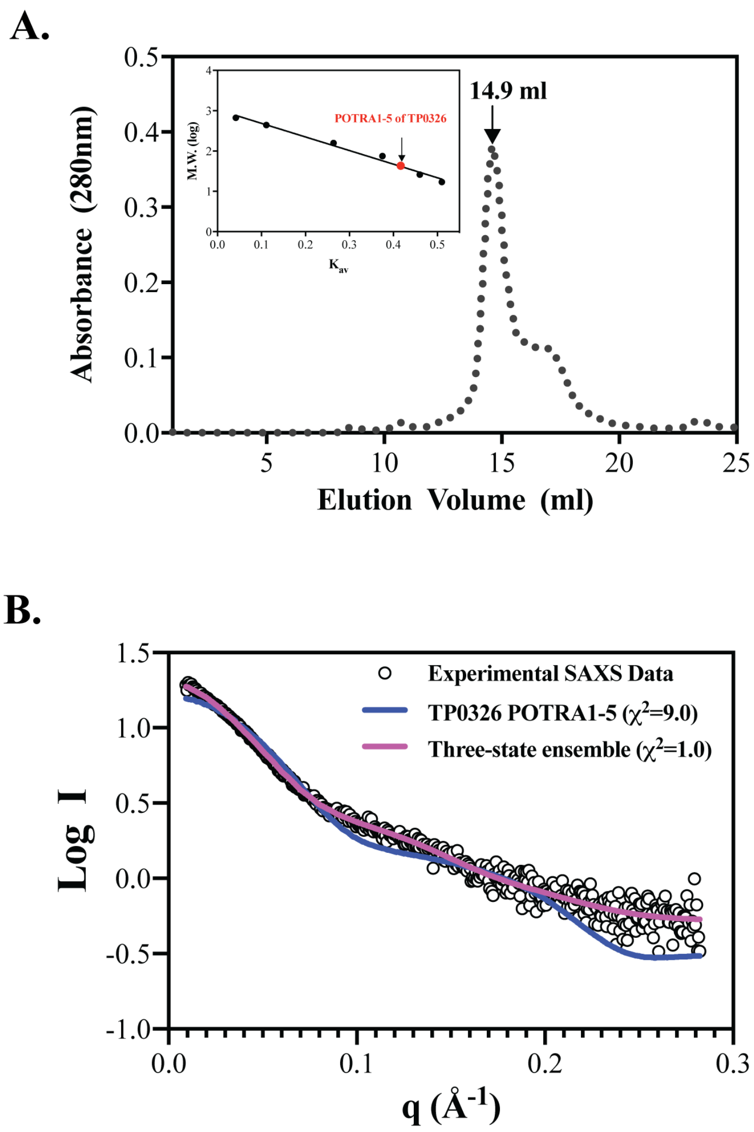

Fig. S2. Purification and SAXS data collection for TP0326 POTRA1-5. A. Size-exclusion chromatography of POTRA1-5 of TP0326 produces a single peak corresponding to a monomer with a molecular weight of ~45 kDa. The inset shows the SEC calibration curve calculated by a linear fit of known molecular weight (M.W.) standards as a function of measured partition coefficients (K_{av}). **B**. Plots show the log of the scattering intensity (I) as a function of momentum transfer ($q = 4\pi\sin(\theta)/\lambda$). The black open circles are SAXS experimental data; the theoretical scattering curves are represented as solid lines. Associated χ^2 values are shown in the parenthesis.

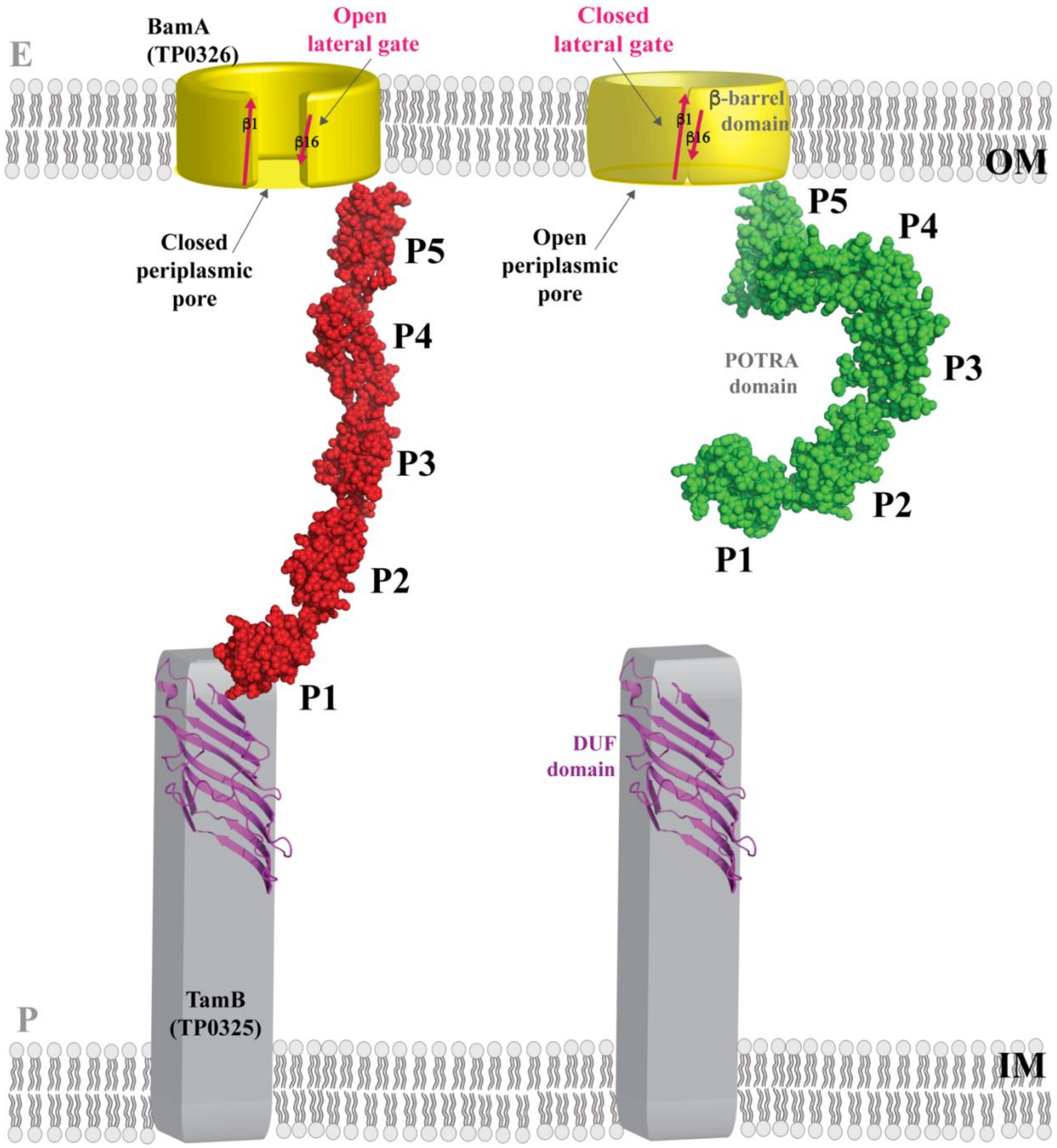

Fig. S3. Proposed working model for POTRA-mediated transfer of nascent OMPs from periplasm to the BamA channel. Extended and bent conformations of POTRA1-5 of TP0326 are shown in red and green spheres, respectively. The DUF domain of TP0325 (TamB) is shown in magenta. E: extracellular surface, OM: outer membrane, P: periplasmic space, IM: inner membrane.

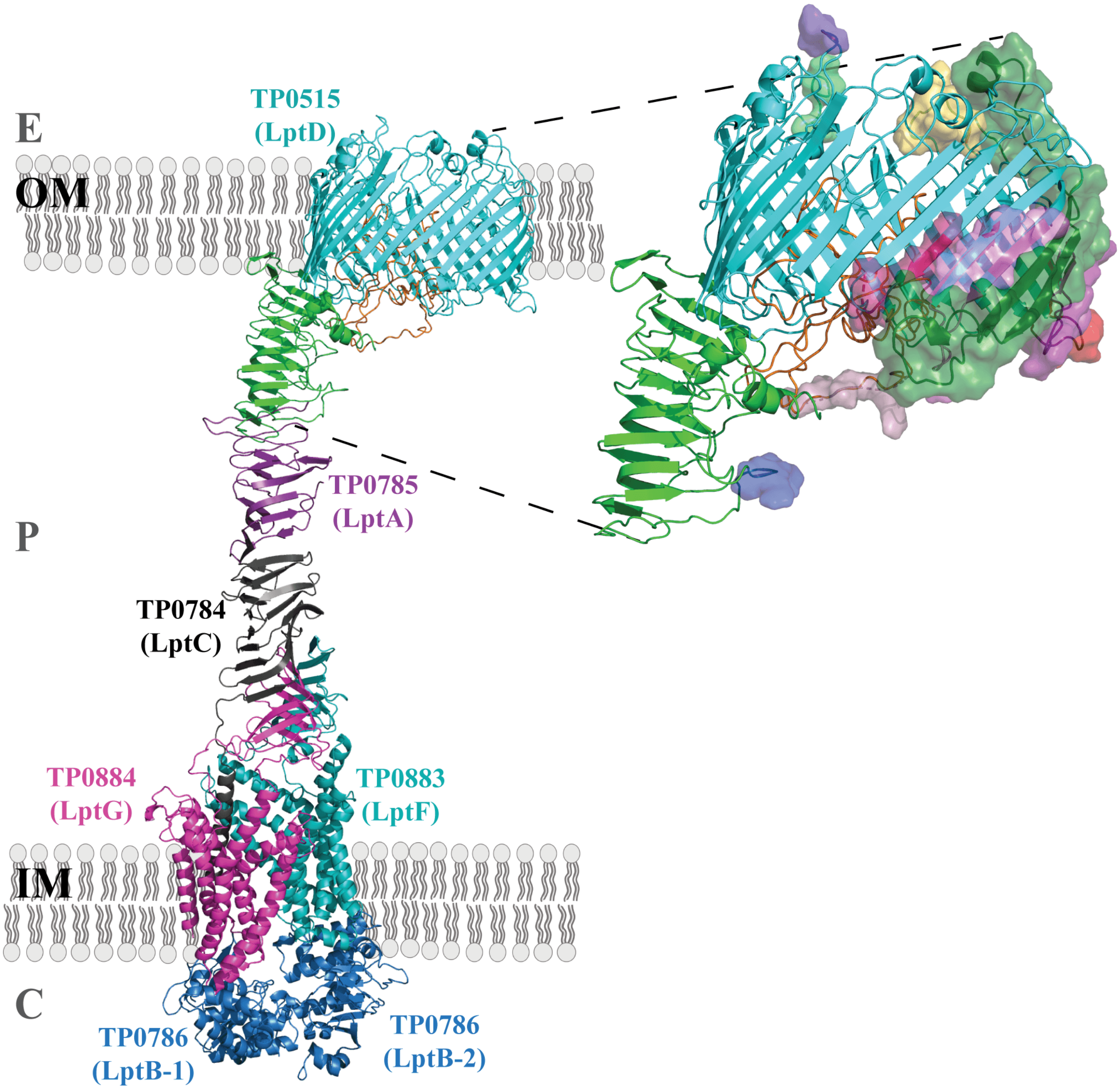

Fig. S4. ***T. pallidum*** **contains orthologs for all components of the LptD complex except LptE.** The ribbon diagram shows the assembled 3D models of LptB2(TP0786)-LptF(TP883)-LptG(TP0884)-LptC(TP0784)-LptA(TP0785)-LptD(TP0515). TP0515 is zoomed in for better clarity of BCEs predicted by DiscoTope 2.0, shown as a transparent surface.

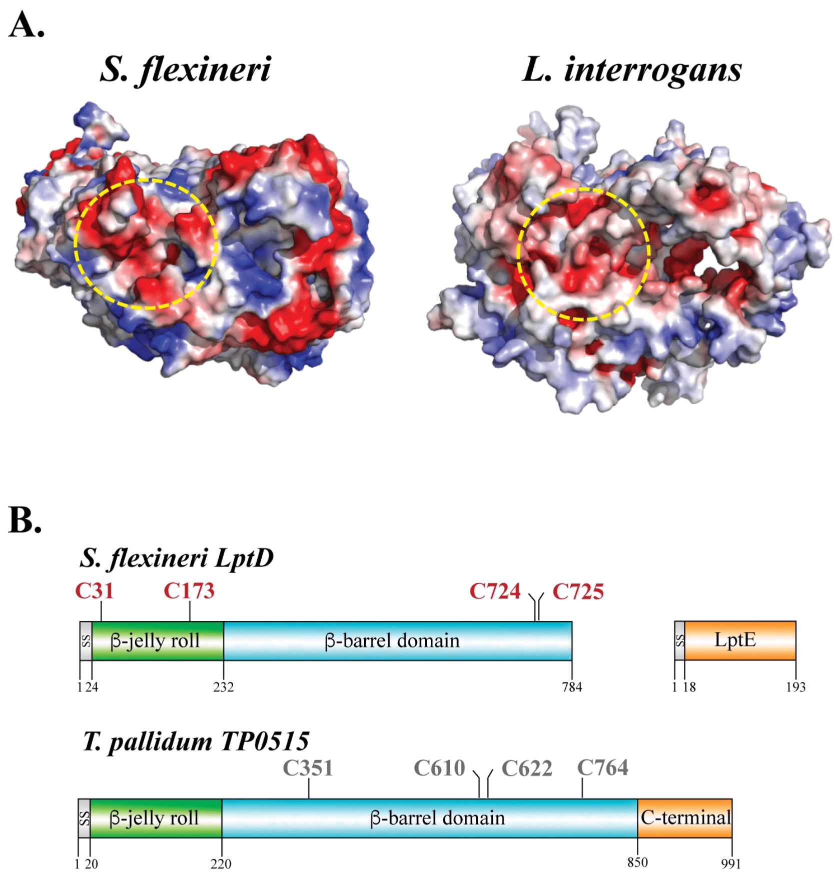

Fig. S5. Electrostatics and domain boundaries of LptDs. A. Comparative electrostatic potentials (same orientation) of the crystal structure of *S. flexineri* LptD (PDB ID: 5JK2) and structural models for LptDs of *L. interrogans* (LIC11458), and *T. pallidum* (TP0515). Shown are the extracellular surfaces of the β-barrel domains. Yellow dashed lines encircle the exit pores. Surfaces are colored according to the local electrostatic potential (−10 kT/e to +10 kT/e), calculated using the ABPS plugin in PyMOL (https://pymol.org/). **B.** Schematic representation of domain boundaries and positions of Cys residues for *S. flexineri* LptD, *S. flexineri* LptE and *T. pallidum* LptD (TP0515).

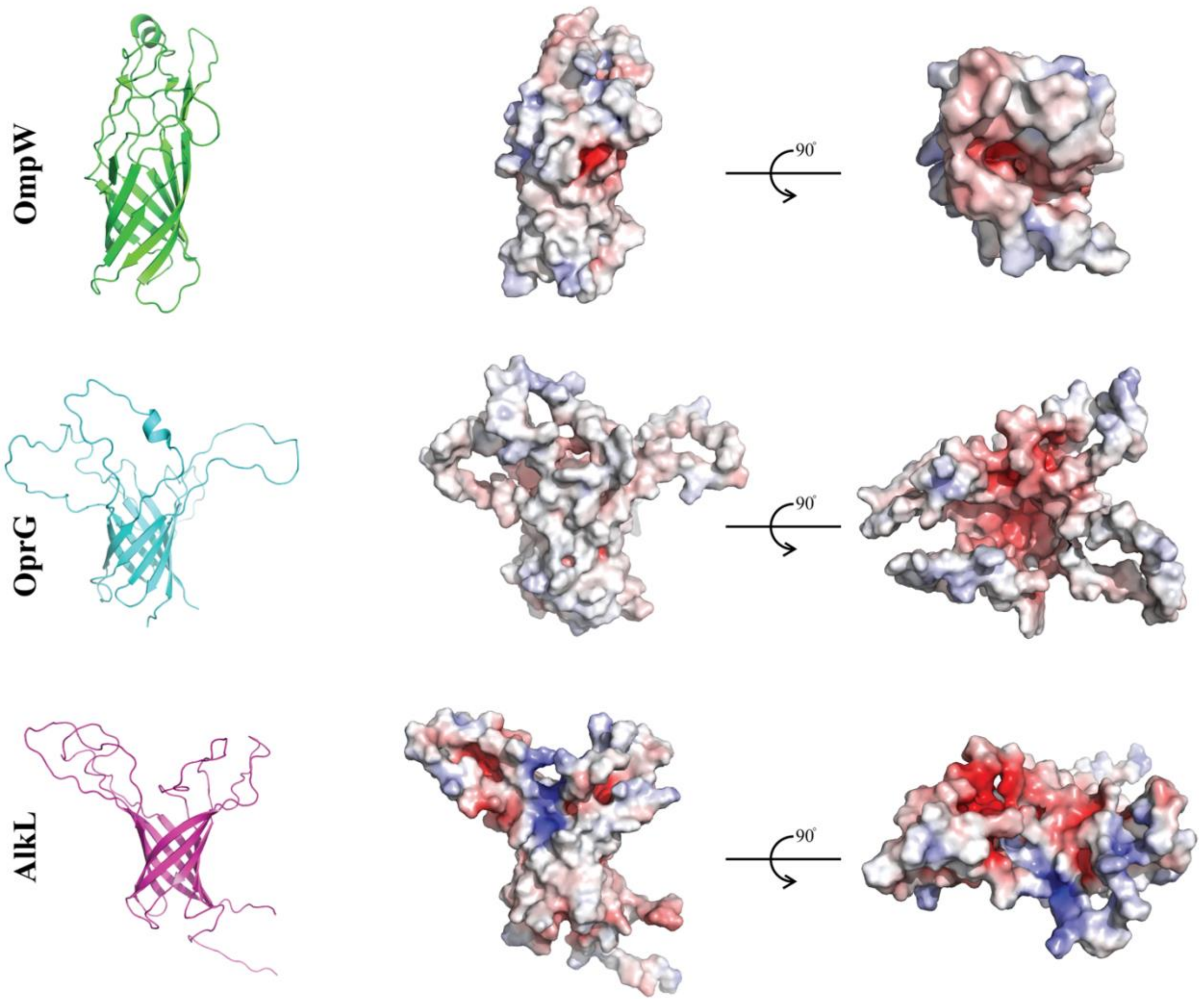

Fig. S6. Cartoon diagrams representing NMR structures of OmpW (PDB ID: 2MHL), OprG (PDB ID: 2N6L), and AlkL (PDB ID: 6QAM) and their electrostatic potentials. All proteins are in the same orientation. The surface is colored according to the local electrostatic potential (−10 kT/e to +10 kT/e), calculated using the ABPS plugin in PyMOL.

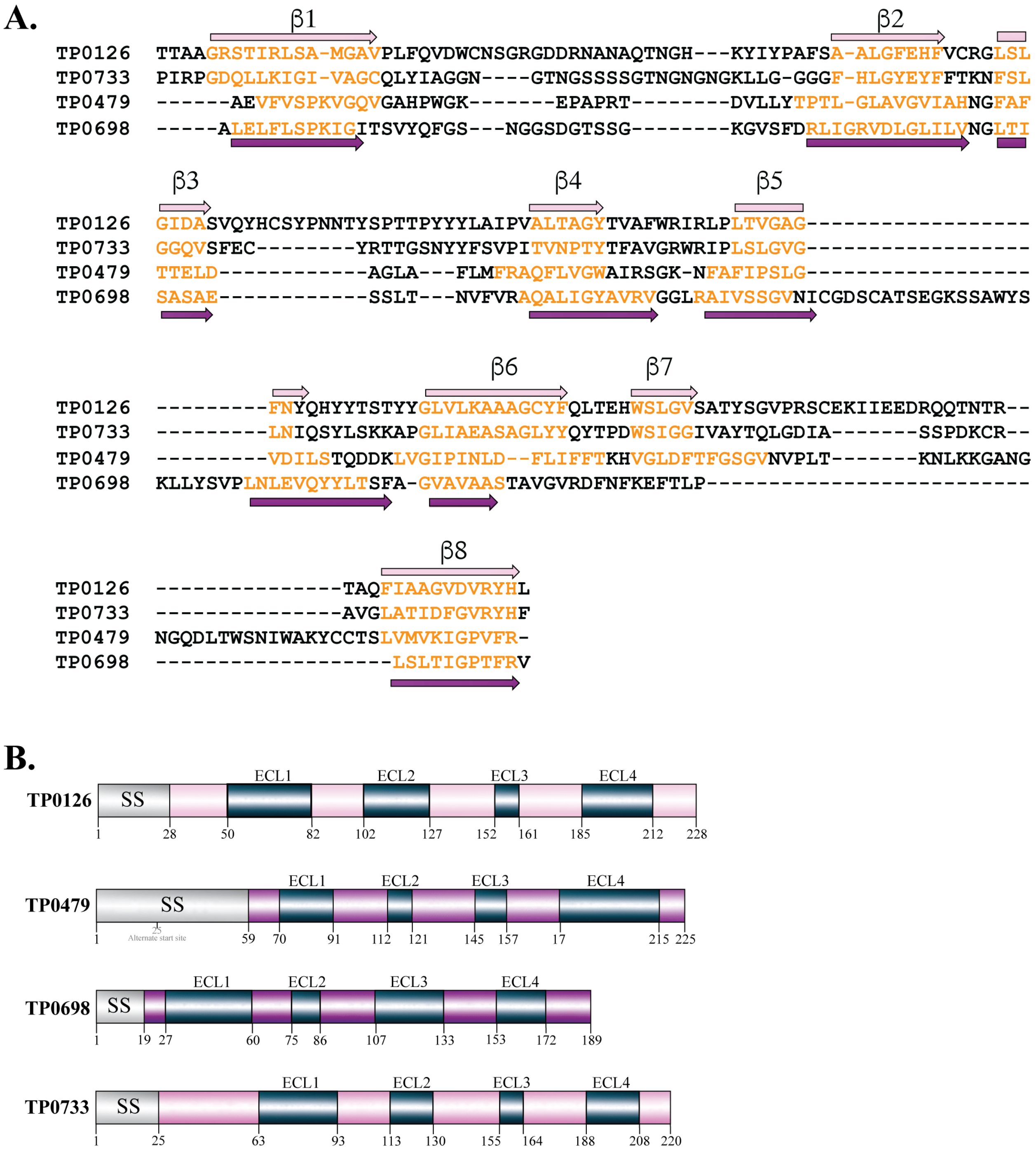

Fig. S7. A. Structure-based sequence alignment of *T. pallidum* 8-stranded barrels. The amino acids of the β-strands are shown in yellow. The β-strands for TP0126 and TP0698 also are depicted as arrows above and below their respective sequences. **B.** Schematic representation of SS (signal sequence) and ECL boundaries for TP0126, TP0479, TP0698 and TP0733.

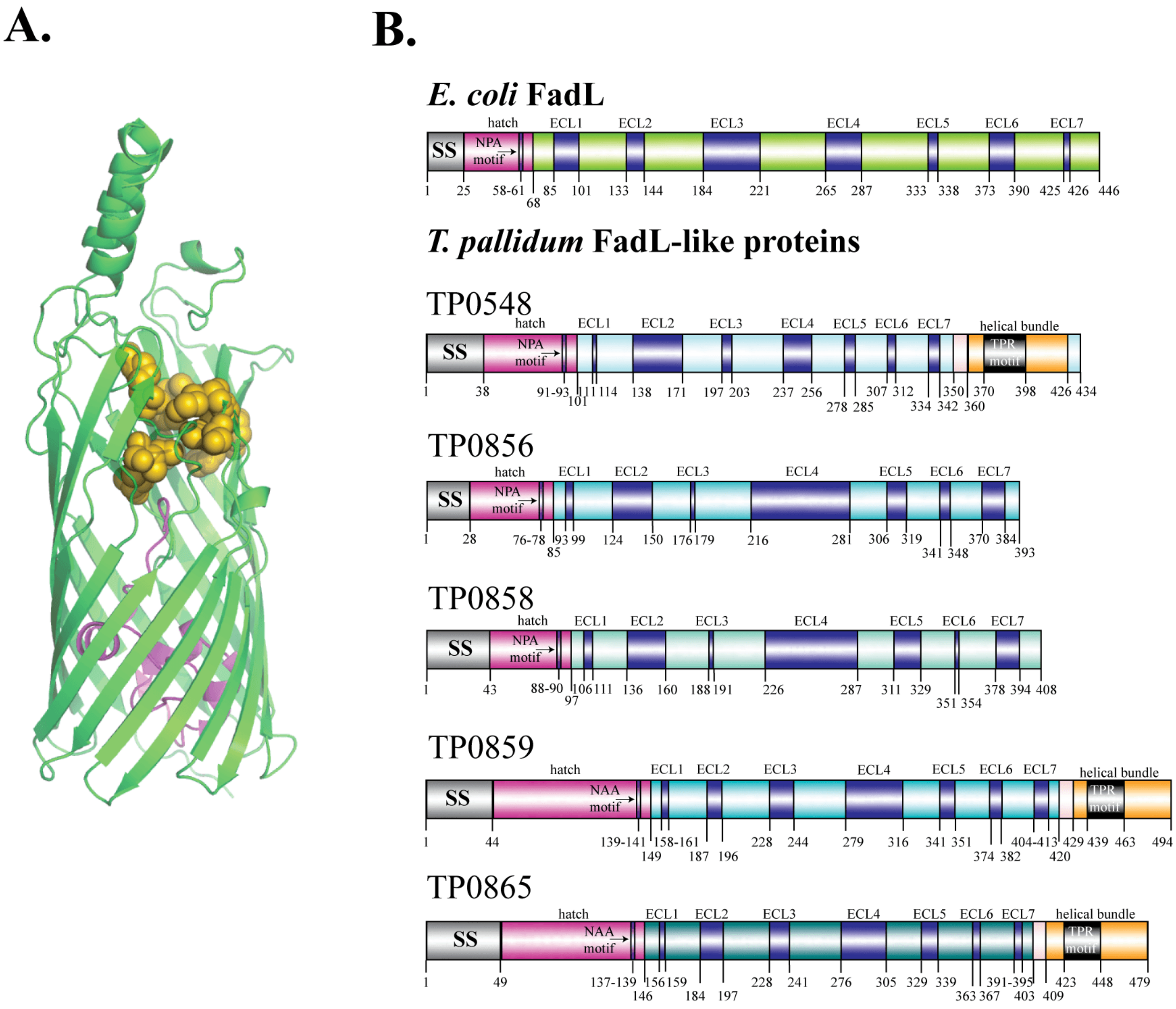

Fig. S8. A. Ribbon diagram of the crystal structure of *E. coli* FadL (PDB ID:1T1L). Residues of the LCFA high-affinity binding site are shown as yellow spheres. **B.** Schematic representation of SS (signal sequences), N-P/A-A motifs and ECL boundaries for *E. coli* FadL and *T. pallidum* FadL-like proteins.

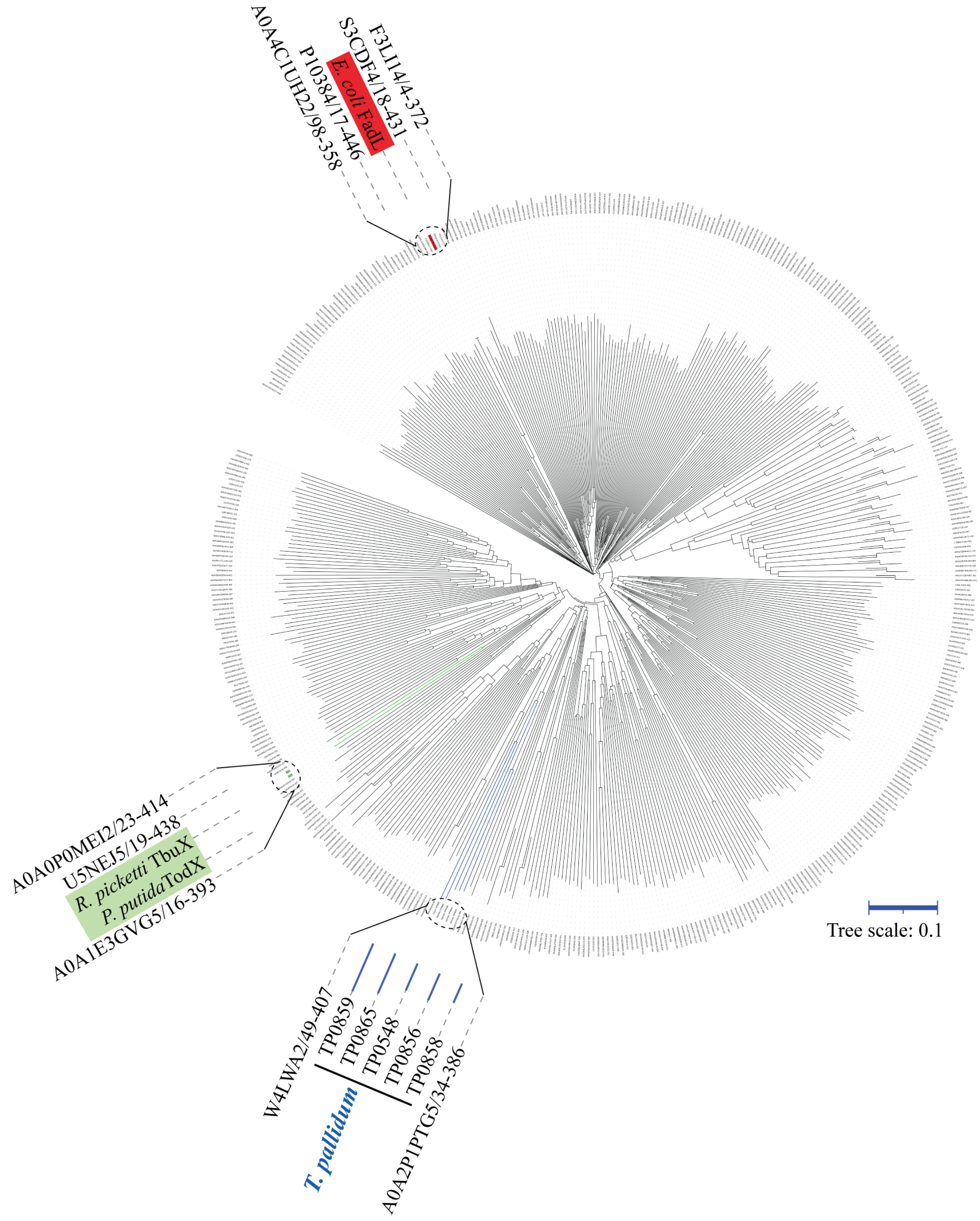

Fig. S9. Phylogenetic analysis of FadL-like proteins. Rooted circular cladogram showing sequence-based phylogenetic relationships between *T. pallidum* FadL orthologs and 415 FadL proteins from the Pfam database. Branches, which represent *E. coli* FadL, TodX/TbuX and *T. pallidum* FadL-like proteins, are zoomed in for clarity.

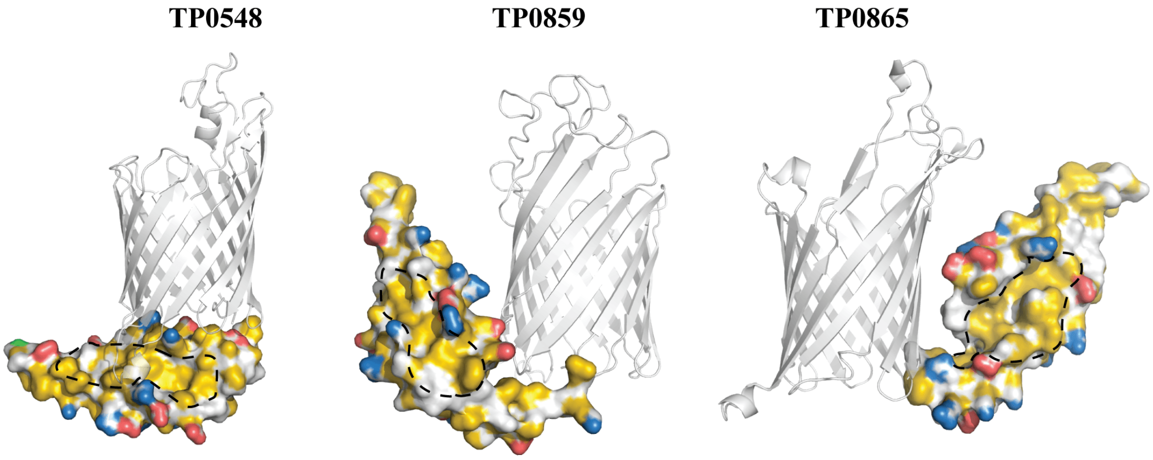

Fig. S10. Hydrophobicity for C-terminal domains of TP0548, TP0859, and TP0865. The dashed ovals represent possible hydrophobic grooves in C-terminal domains. β-barrel domains, without hatch regions, are shown as ribbon diagrams. The hydrophobic surfaces are colored according to the YRB highlighting scheme (yellow - hydrocarbon groups without polar substitutions; red - negatively charged oxygens of glutamate and aspartate; blue - nitrogens of positively charged functional groups of lysines and arginines; and white - all remaining atoms, including the polar backbone).

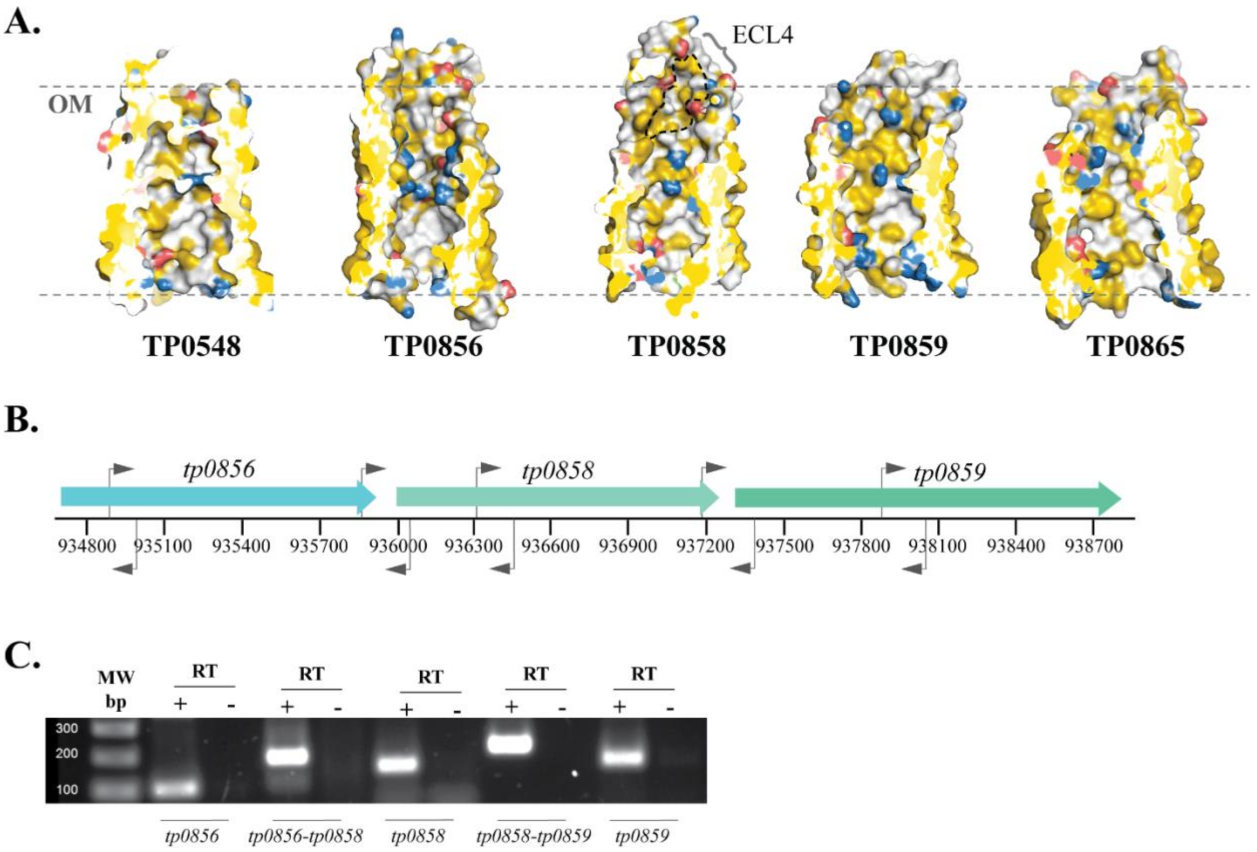

Fig. S11. A. Hydrophobicity within the channels of *T. pallidum* FadL-like proteins TP0548, TP0856, TP0858, TP0859, and TP0865. Surfaces are colored according to the same YRB highlighting scheme used in Fig. S10. The dashed line in TP0858 represents a hydrophobic patch in ECL4. **B.** Schematic depiction of the *tp0856-tp0859* operon. Arrows indicate the locations of primers (Table S9) used for PCR amplification of *tp0856*, *tp0858*, *tp0859*, and intergenic regions. **C.** RT-PCR was performed on *T. pallidum* RNA using the primer pairs listed in **Table S9**. Lane MW, DNA molecular size markers. Lanes RT -: PCR with the indicated primer pairs and RNA as the template but without RT. Lanes RT +: PCR with the indicated primer pairs and RNA as the template but with RT.

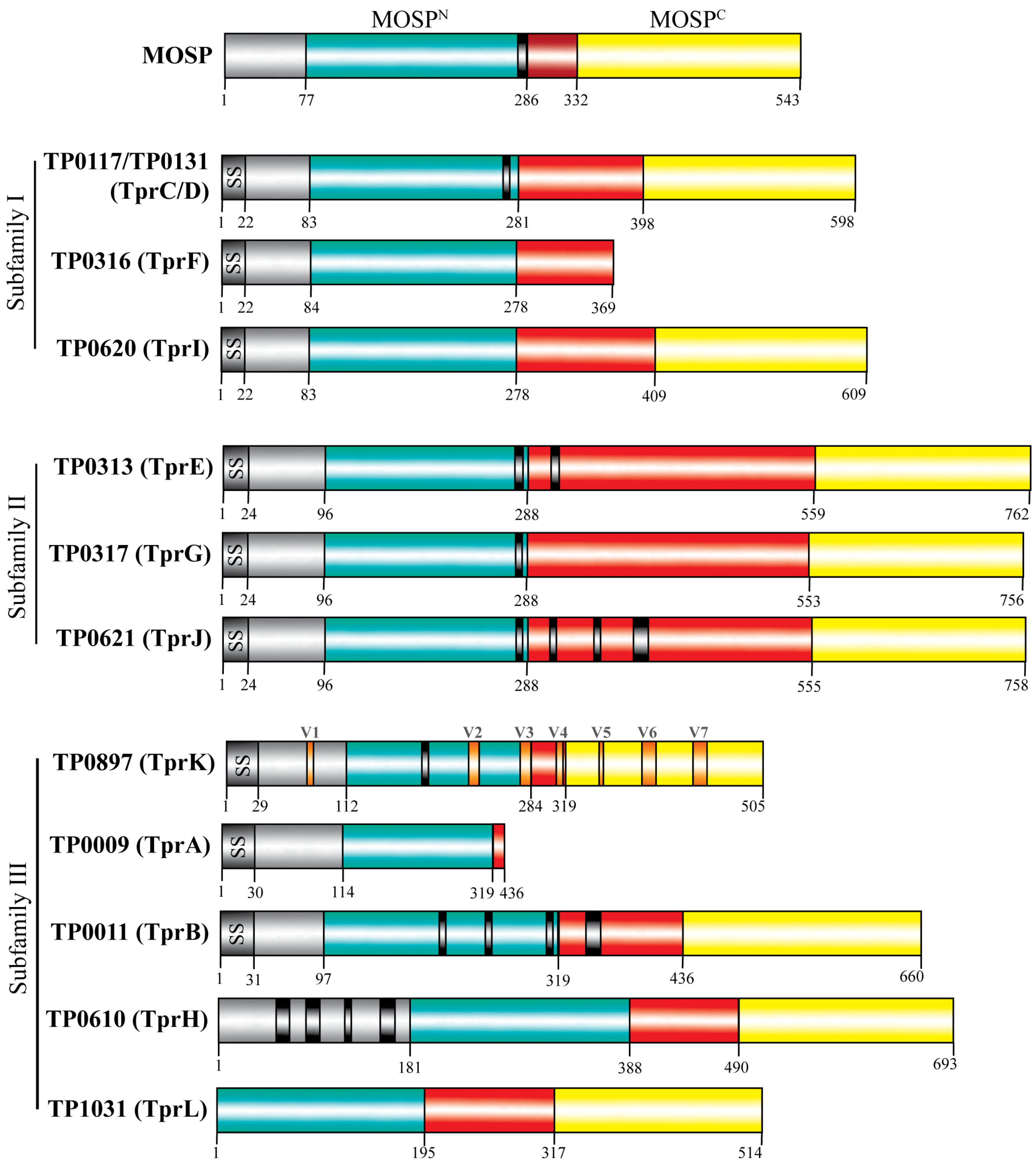

Fig. S12. Domain boundaries of *T. denticola* MOSP and *T. pallidum* Tpr proteins from the Pfam database. The signal sequences (SS) and extreme amino-terminal stretches are shown in dark and light grey, respectively. MOSP^N and MOSP^C domains are colored in cyan and yellow, respectively; CVRs are shown in red. MoRFs are shown in black. The seven variable regions of TprK are shown in orange (1).

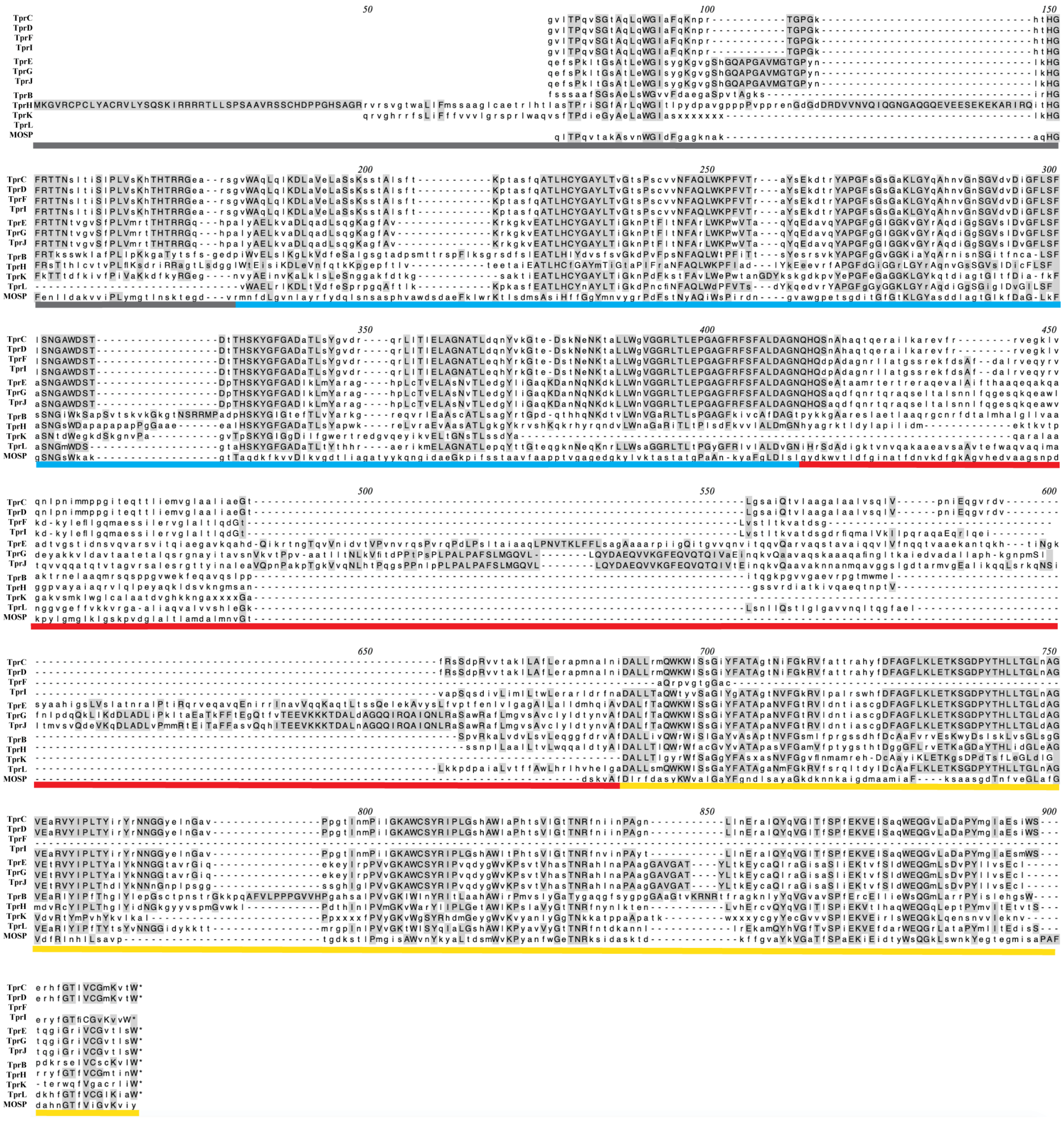

Fig. S13. MSA of each Tpr domain. The extreme amino-terminal stretch is highlighted by the grey line. $MOSP^N$ and $MOSP^C$ domains, according to the Pfam database, are indicated by cyan and yellow lines, respectively; the central variable regions are indicated by the red line. Grey shading represents 51% consensus identity among amino acid residues; lower-case letters highlight mismatched residues in that location.

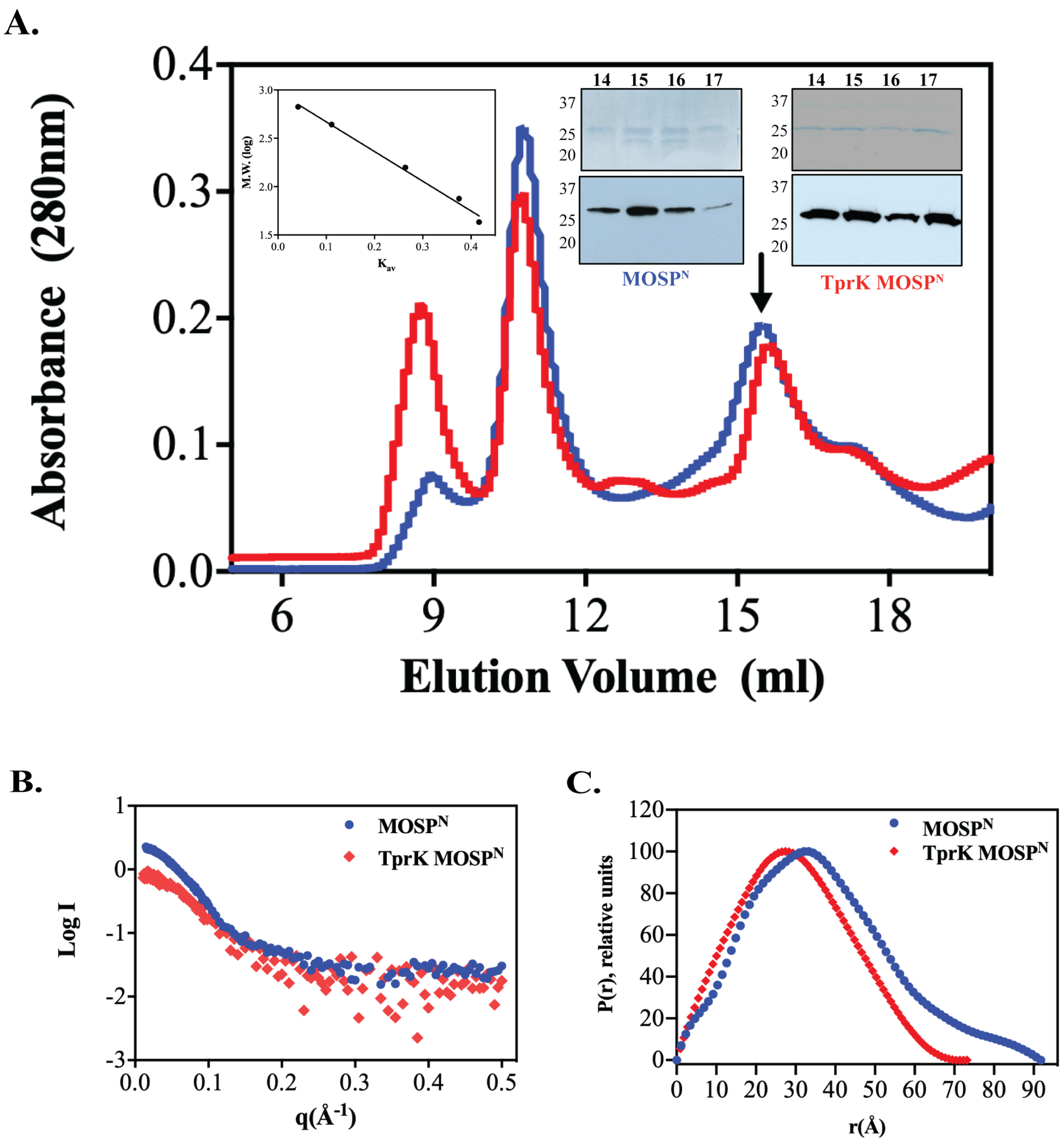

Fig. S14. Purification and SAXS data for MOSP^{N} domains of *T. denticola* MOSP and TprK. **A.** SEC profiles of MOSP^{N} domains of MOSP (blue line) and TprK (red line) collected by monitoring absorbance at 280 nm. Left inset: SEC calibration curves calculated by a linear fit of known molecular weight (M.W.) standards as a function of measured partition coefficients (K_{av}). Right inset: SDS-PAGE of eluded fractions of MOSP^{N} domains of MOSP and TprK. **B.** Solution scattering curves of MOSP^{N} domains of MOSP and TprK. **C.** Comparison of the P(r) functions calculated from the experimental data of the two MOSP^{N} domains.

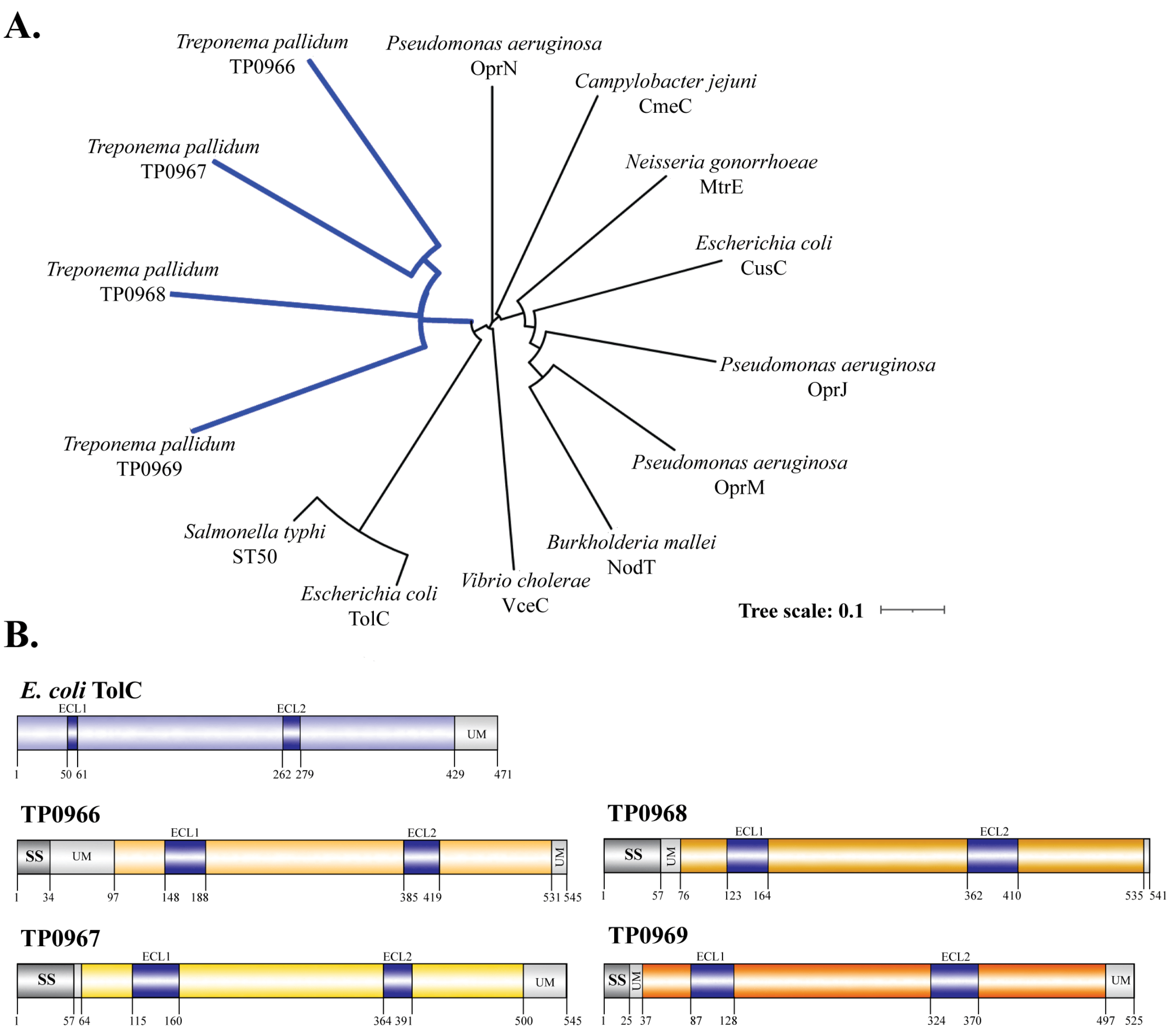

Fig. S15. Phylogenetic analysis and ECL boundaries of *T. pallidum* OMFs. A. The rooted circular cladogram shows the sequence-based phylogenetic relationships between *T. pallidum* OMFs (TP0966, TP0967, TP0968, and TP0969) and characteristics OMFs from other Gram-negative bacteria. **B.** Linear schematic representation of *E. coli* TolC and *T. pallidum* OMF monomers. ECLs are colored in blue. SS and UM represent signal sequences and unmodeled regions, respectively.

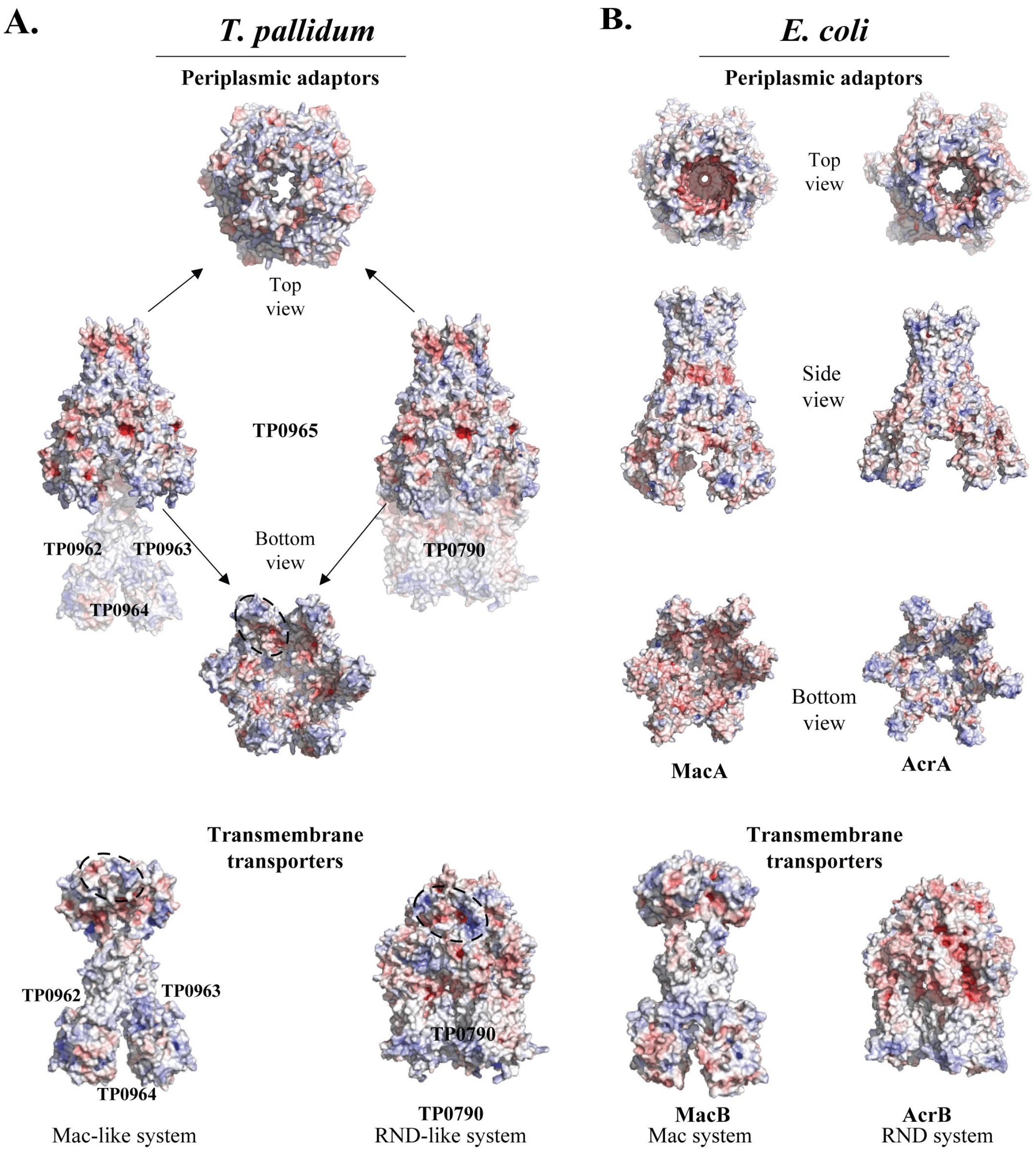

Fig. S16. Electrostatics of periplasmic adaptors and TM transporters. A. Electrostatic potentials of TP0965 (ortholog of periplasmic adaptor), TP0962/TP0963/TP0964 (Mac-like transporter), and TP0790 (RND-like transporter). Dashed lines represent one patch of mixed electrostatic charges. **B.** Electrostatics of *E. coli* periplasmic adaptors (MacA, AcrA) and transmembrane transporters (MacB and AcrB). Surfaces are colored according to the local electrostatic potential ranging from blue (+10 kT/e) to red (−10 kT/e).

Table S1: Abbreviations

Acronym	Definition
ABC	ATP-binding cassette
Acr	Acridine resistance protein
AMP	Anti-microbial peptide
BAM	b-barrel assembly machine
BCE	B-cell epitope
Caro	*Carbapenem* -associated outer membrane protein
CD	Circular dichroism spectroscopy
CM	Cytoplasmic membrane
Cryo-EM	Cryoelectron microscopy
CVR	Central variable region
DUF	Domain of unknown function
ECL	Extracellular loop
ECM	Extracellular matrix
FadL	Long-chain fatty acid transport protein
LCFA	Long chain fatty acid
LPS	Lipopolysaccharide
Lpt	LPS transport
MAC	*Mycobacterium avium-M. intracellulare* complex
MoRF	Molecular recognition features
MSA	Multiple sequence alignment
MOSP	Major outer sheath protein
$MOSP^{C}$	C-terminal portion of MOSP
$MOSP^{N}$	N-terminal portion of MOSP
Mtr	Multiple transferable resistance system
NBD	Nucleotide binding domain
OM	Outer membrane
OMF	Outer membrane factor
OMP	Outer membrane protein
OMPeome	Repertoire of *Treponema pallidum* OMPs
POTRA	Polypeptide transport-associated domain
RND	Resistance-nodulation-cell division
SAXS	Small-angle X-ray scattering
TAM	Translocation and assebmly module
TbuX	Gene x of toluene *m* -monooxygenation pathway
TM	Transmembrane
TodX	Gene x of toluene dioxygenation pathway
TPR	Tetratricopeptide repeat
Tpr	*Treponema pallidum* repeat protein
TRAP transporter	Tripartite ATP-independent periplasmic transporters

Table S2: Predicted BCEs of *T. pallidum* TP0515

TP0515

BCE1

Region	Residue	Score
N-terminal domain	THR37	-3.43
	ARG38	0.82
	LEU39	-0.033
	SER40	-3.088

BCE2

Region	Residue	Score
ECL3	GLY318	-3.501
	ASN319	-2.304
	GLY320	-2.257
	TRY322	-3.618

BCE3

Region	Residue	Score
Inner loop	VAL439	-2.839
	HIS440	0.057
	PHE441	0.114
	ASN442	2.705
	SER443	4.124
	LYS444	3.172
	SER445	4.507
	ASP446	5.742
	SER447	5.43
	LYS448	5.279
	LYS449	3.474
	ASN450	-1.372
	ASN451	-2.513

BCE4

Region	Residue	Score
ECL5	TYR460	-2.271
	PRO461	-3.55
	HIS462	-1.319
	SER463	2.312
	MET464	2.133
	GLU465	-0.598
	SER466	-2.296
	ARG467	-1.539

BCE5

Region	Residue	Score
Inner loop	GLY474	-2.726
	THR475	-0.303
	LEU476	0.327
	PHE477	3.005
	SER478	4.731
	HIS479	7.563
	VAL480	6.071
	TRP481	5.303
	GLU482	6.841
	ARG483	7.483
	GLN484	8.999
	LYS485	9.403
	SER486	8.978
	GLN487	7.438
	GLN488	6.655
	LYS489	5.221
	GLU490	5.816
	SER491	2.876
	TYR492	2.373
	ALA493	-0.68
	PRO494	1.708
	LYS495	1.015
	GLU496	0.237
	ILE497	-1.882
	ARG498	-0.189

BCE6

Region	Residue	Score
ECL6	ASP507	-2.837
	GLY508	-1.748
	LEU509	-2.154
	SER510	-3.521

BCE7

Region	Residue	Score
β-strand 11, ECL7, and β-strand 12	GLY513	-3.315
	SER514	-2.332
	PRO515	-2.131
	PRO516	-0.262
	GLU517	1.083
	GLN518	0.925
	SER519	2.78
	PRO520	2.594
	ALA521	0.477
	VAL522	1.173
	SER523	2.143
	LYS524	1.591
	GLU525	-0.457
	ASN526	2.615
	SER527	-0.302
	GLU528	1.347
	THR529	-1.709
	ASP530	0.257
	SER531	0.526
	THR532	-0.049
	PHE533	-2.839
	ASP534	-2.074
	PHE536	-0.718
	MET537	-2.83
	PRO538	0.415
	GLU539	-2.69
	PHE540	1.563
	ARG541	0.639
	GLU542	3.735
	GLU543	3.722
	ASN544	5.576
	GLU545	4.456
	ARG546	7.69
	ARG547	6.963
	THR548	5.838
	GLY549	4.173
	THR550	3.976
	ASP551	3.807
	HIS552	0.519
	ALA553	1.95
	TYR554	1.166
	VAL555	0.659
	PHE556	0.633
	THR557	0.531
	ARG558	0.404
	TYR559	0.143
	ALA560	0.889
	LEU561	-1.055
	ASP562	1.101
	TYR563	0.382
	LYS564	0.704
	GLY565	1.313
	LYS566	3.788
	GLY567	2.531
	ASP568	4.11
	ILE569	4.269
	VAL570	4.073
	TYR571	5.652
	ASP572	4.694
	ALA573	3.962
	GLY574	0.763
	PHE575	1.986
	ASN576	0.725
	HIS577	1.586
	GLY578	-2.332
	SER579	-1.274
	TRY580	-2.88
	ASP581	-2.63

BCE8

Region	Residue	Score
Inner loop	CYS623	0.067
	THR624	-2.829
	LEU625	0.999
	GLN626	0.415
	HIS627	3.758
	ARG628	5.042
	TYR629	3.528
	PRO630	5.872
	ILE631	3.898
	HIS632	4.553
	SER633	5.654
	GLN634	6.79
	PRO635	3.781
	PRO636	5.856
	ALA637	2.95
	LYS638	1.641
	GLN639	1.468
	ASN640	0.882
	GLY641	-2.271
	PHE642	-2.307

BCE9

Region	Residue	Score
Inner loop	PRO676	-1.273
	VAL677	-3.64
	LEU678	-1.586
	TRY679	-1.744
	ASP680	-0.903
	SER681	1.185
	SER682	-2.879
	PRO684	-2.87

BCE10

Region	Residue	Score
ECL12	ASN807	-0.686
	ASN808	-0.779
	GLY809	-0.634

BCE11

Region	Residue	Score
C-terminal extension	MET975	-2.302
	ARG977	0.103
	THR978	-3.57
	ARG979	-2.451
	ARG981	-2.809
	PRO984	-2.473

a. Discontinuous BCEs were predicted from 3D models using a threshold of -3.7 in the DiscoTope 2.0 Server (2). The score for BCE is calculated as a combination of propensity scores of amino acids in spatial proximity and contact numbers. The residue contact number is the number of Cα atoms in the antigen within a distance of 10 Å (3).

Table S3: Predicted BCEs of *T. pallidum* eight-stranded β-barrel OMPs

TP0126

Loop	Residue	Score
**BCE1**		
ECL1	GLY32	-3.025
ECL1	ARG33	2.091
ECL1	GLY34	3.579
ECL1	ASP35	3.399
ECL1	ASP36	1.568
ECL1	ARG37	0.722
ECL1	ASN38	3.821
ECL1	ALA39	3.24
ECL1	ALN40	0.772
ECL1	ALA41	2.893
ECL1	GLN42	-1.243
ECL1	THR43	2.228
ECL1	ASN44	1.36
ECL1	GLY45	-3.604
ECL1	HIS46	-1.699
**BCE2**		
ECL2	PRO82	-3.215
ECL2	ASN83	-1.162
ECL2	ASN84	1.932
ECL2	THR85	2.71
ECL2	TYR86	-0.055
ECL2	SER87	2.923
ECL2	PRO88	1.535
ECL2	THR89	-0.181
ECL2	THR90	0.17
ECL2	PRO91	1.195
ECL2	TYR92	-0.041
ECL2	TYR93	-2.838
**BCE3**		
ECL3	THR128	-2.369
ECL3	THR129	-3.329
ECL3	SER130	-2.497
**BCE4**		
ECL4	GLU167	-0.57
ECL4	LYS168	0.881
ECL4	ILE169	3.105
ECL4	ILE170	3.049
ECL4	GLU171	2.731
ECL4	GLU172	2.435
ECL4	ASP173	2.705
ECL4	ARG174	3.865
ECL4	GLN175	2.242
ECL4	GLN176	0.7
ECL4	THR177	-0.636
ECL4	ASN178	0.193

TP0733

Loop	Residue	Score
**BCE1**		
Inner loop	PRO21	-0.68
Inner loop	ILE22	-2.439
Inner loop	ARG23	-2.906
Inner loop	PRO24	-1.972
Inner loop	GLY25	-2.23
**BCE2**		
ECL1	THR55	-1.013
ECL1	ASN56	-2.541
ECL1	GLY57	-2.819
ECL1	SER58	-2.777
**BCE3**		
Inner loop	GLN152	-3.616
Inner loop	THR154	-0.2
Inner loop	PRO155	0.829
Inner loop	ASP156	0.52
Inner loop	THP157	-3.661
**BCE4**		
ECL4	SER174	-3.56
ECL4	PRO175	-2.543
ECL4	ASP176	-1.771

TP0479

Loop	Residue	Score
**BCE1**		
ECL1	GLY18	-1.941
ECL1	LYS19	-0.077
ECL1	GLU20	1.061
ECL1	PRO21	1.001
ECL1	ALA22	0.01
ECL1	PRO23	0.38
ECL1	ARG24	-0.731
ECL1	THR25	-0.873
ECL1	ASP26	-1.895
**BCE2**		
ECL4	PRO125	-1.548
ECL4	LEU126	-0.215
ECL4	THR127	-0.008
ECL4	LYS128	0.881
ECL4	ASN129	1.287
ECL4	LEU130	1.589
ECL4	LYS131	0.493
ECL4	LYS132	0.48
ECL4	GLY133	-1.547
ECL4	ASN135	-0.459
ECL4	GLY136	-2.693
ECL4	ASN137	-1.327
ECL4	GLY138	-2.609

TP0698

Loop	Residue	Score
**BCE1**		
ECL1	ASN22	-3.684
ECL1	GLY24	-3.667
ECL1	SER25	-1.469
ECL1	ASP26	-1.11
ECL1	GLY27	0.863
ECL1	THR28	-0.224
ECL1	SER29	-0.208
ECL1	SER30	-0.335
ECL1	GLY31	-0.735
ECL1	LYS32	-0.616
ECL1	GLY33	-1.837
ECL1	VAL34	-1.626
ECL1	SER35	-1.125
ECL1	PHE36	-1.638
**BCE2**		
ECL3	GLU102	-3.505
ECL3	GLY103	-2.551
ECL3	SER105	-2.837
ECL3	SER106	-3.026

Table S4: Sequence similarity of *T. pallidum* FadL-like OMPs

	% Sequence Similarity				
	**TP0548**	**TP0856**	**TP0858**	**TP0859**	**TP0865**
**TP0548**	100				
**TP0856**	34.9	100			
**TP0858**	33.5	72.0	100		
**TP0859**	41.0	30.2	30.6	100	
**TP0865**	42.5	30.8	30.7	60.3	100

Table S5: Predicted BCEs of *T. pallidum* FadL-like OMPs

TP0548

BCE	Region	Residue	Score
BCE1	N-terminal extension	SER46	-0.851
		GLY47	-1.755
		SER48	0.636
		GLY49	-1.576
		SER50	2.063
		ASP51	-1.082
		GLY52	-2.318
BCE2	ECL2	GLU140	-1.485
		SER141	-0.270
		ASP142	-3.474
		LYS144	-0.991
		SER145	-2.044
		PHE147	-2.838
		GLY148	0.427
		GLY149	-0.032
		ASN150	3.421
		SER151	3.860
		GLY152	4.049
		GLY153	3.245
		ASN154	4.064
		LYS155	0.0818
		ASN156	2.041
		GLY157	0.154
		GLY158	-1.216
		HIS159	-3.254
		GLN160	-0.815
		GLY161	1.490
		LYS162	3.859
		GLN163	2.319
		GLY164	2.487
		LYS165	1.665
		GLY166	-1.747
		PHE167	-2.610
BCE3	ECL4	ASN246	-3.006
		SER247	-3.408
		CYS248	-2.033
BCE4	CT extension	PRO354	-2.647
		GLY356	-1.117
		GLY357	-0.628
		GLN359	-3.132

TP0856

BCE	Region	Residue	Score
BCE1	ECL1	PHE96	-0.142
		ASN97	1.750
		ASN98	0.150
BCE2	ECL2	PRO124	-3.61
		GLU125	-0.035
		SER126	-0.245
		GLY127	-0.202
		PHE128	-3.124
		ASN129	-1.897
BCE3	ECL7	THR372	-2.191
		GLY373	0.751
		ASP374	1.211
		GLU375	2.246
		GLN376	4.353
		GLN377	3.332
		GLY378	-2.184
		THR379	-3.198

TP0858

BCE	Region	Residue	Score
BCE1	N-terminal extension	ALA43	-3.115
		ALA45	-2.786
		LYS46	-1.059
		PRO47	-3.452
		LYS48	-2.038
		LYS49	2.166
		GLY50	-0.681
		GLN51	-2.296
		MET52	-1.919

TP0859

BCE	Region	Residue	Score
BCE1	N-terminal extension	ARG76	-1.778
		SER77	-2.648
		ARG78	-3.189
		GLY79	-0.326
		GLY80	0.256
BCE2	Hatch	THR94	-0.742
		ALA95	1.894
		GLN96	3.482
		LYS97	4.053
		PRO98	0.472
		LYS99	1.746
		VAL100	-1.282
		MET101	-3.116
		ALA102	-2.17
BCE3	ECL2	ASP188	0.0000
		MET189	0.341
		SER190	3.630
		SER191	4.849
		LYS192	2.871
		THR193	-0.706
		THR194	0.025
		GLY195	-2.864
BCE4	BCE3	HIS227	-2.68
		THR228	-1.051
		ARG229	2.865
		GLY230	5.980
		GLY231	5.847
		GLY232	6.779
		SER233	5.357
		SER234	2.141
		GLN235	6.172
		SER236	4.492
		LYS237	7.159
		SER238	7.155
		SER239	8.182
		ASN240	8.180
		GLY241	8.010
		LYS242	7.576
		GLU243	3.924
		ASN244	2.154
		HIS245	0.337
BCE5	ECL5	THR288	-1.092
		ASN289	1.978
		LEU290	-2.729
		HIS291	0.739
		GLY292	2.312
		ASN293	4.690
		ASN294	5.634
		GLY295	7.032
		ALA296	4.927
		GLY297	4.261
		GLY298	0.699
		SER299	4.906
		GLY300	3.756
		GLY301	2.472
		GLY302	-0.474
		GLY303	1.081
		GLY304	2.547
		GLY305	2.661
		ASN306	6.313
		GLY307	4.383
		ASP308	3.329
		GLY309	0.628
		LYS310	-0.481
BCE6	C-terminal extension	PRO427	-0.413
		GLU428	0.207
		GLN429	-0.859
		PRO430	0.296
		LEU431	-2.135
		TRP432	-3.474
		GLN433	-1.533
		GLU434	-3.257
BCE7	C-terminal extension	ALA448	-3.05
		GLN449	-2.151
		ARG450	-2.835
		ALA452	-2.554
		GLU453	-3.379
		ALA456	-3.679
		ARG460	-3.032
		GLN463	-2.864
		GLN464	-2.963
		GLY465	-1.856
		SER467	-3.061
BCE8	C-terminal extension	GLN486	-2.576
		HIS489	-1.649
		ASP490	-1.005
		PHE491	-1.539
		ASN492	0.177
		ILE493	-1.282
		PHE494	-0.698

TP0865

BCE	Region	Residue	Score
BCE1	N-terminal extension	GLU99	-2.646
		LYS100	-2.73
		GLN101	-2.827
		LYS102	-0.267
		GLY103	1.178
		VAL104	-1.18
		GLY105	0.377
BCE2	ECL3	SER229	-3.119
		ALA230	-2.363
		GLY231	-0.769
		GLY232	2.32
		GLU233	-0.870
BCE3	ECL3	ASN238	-3.382
		GLN239	0.066
		GLY240	0.887
		GLY241	-0.072
		LYS242	-3.682
BCE4	periplasmic loop	PHE263	-3.448
		GLY264	-1.427
		SER265	-3.027
BCE5	ECL4	GLU284	-3.575
		ASP286	-1.117
		ALA287	0.997
		SER288	0.297
		ASN289	1.928
		SER290	0.446
		GLY291	2.435
		SER292	1.708
		SER293	1.226
		MET294	-0.332
		SER295	-0.394
		GLY296	-2.32
		GLY297	-1.701
		ARG298	-1.881
BCE6	CT extension	ALA335	-2.473
		ASP336	-3.131
		ASN337	-3.415
BCE7	C-terminal extension	ARG409	-3.46
		ASP410	-1.217
		ARG411	-3.248
		LYS413	-2.396
		HIS414	-1.868
		LYS417	-2.133
		GLU418	-3.516
		TYR420	-3.255
		THR421	-1.169
		SER422	-2.739
		TYR423	-2.92
		LEU424	-1.558
		ARG425	-2.71
		LEU427	-2.677
		ARG428	-2.299
		ASP431	-2.449
		ALA432	-3.445

Table S6: Sequence similarity matrix of each *T. pallidum* Tpr domain.

**NT region**		**Subfamily I**				**Subfamily II**			**Subfamily III**				
		TP0117	TP0131	TP0317	TP0620	TP0313	TP0317	TP0621	TP0011	TP0897	TP0610	TP1031	MOSP
**Subfamily I**	TP0117	100											
	TP0131	100	100										
	TP0317	98.5	98.5	100									
	TP0620	99.0	99.0	99.5	100								
**Subfamily II**	TP0313	70.6	70.6	70.4	70.1	100							
	TP0317	70.6	70.6	70.4	70.1	100	100						
	TP0621	70.6	70.6	70.4	70.1	100	100	100					
**Subfamily III**	TP0011	50.9	50.9	50.7	50.4	53.8	53.8	53.8	100				
	TP0610	63.6	63.6	63.5	63.2	61.1	61.1	61.1	55.6	100			
	TP0897	38.4	38.4	38.6	38.4	41.9	41.9	41.9	35.7	40.0	100		
	TP1031	73.1	73.1	73.0	72.6	69.4	69.4	69.4	54.2	68.8	41.7	100	
	MOSP	31.9	31.9	32.1	31.9	34.0	34.0	34.0	31.9	29.0	28.4	34.0	100

**CVR region**		**Subfamily I**				**Subfamily II**			**Subfamily III**				
		TP0117	TP0131	TP0317	TP0620	TP0313	TP0317	TP0621	TP0011	TP0897	TP0610	TP1031	MOSP
**Subfamily I**	TP0117	100											
	TP0131	100	100										
	TP0317	29.8	29.8	100									
	TP0620	43.1	43.1	63.8	100								
**Subfamily II**	TP0313	13.7	13.7	10.0	12.6	100							
	TP0317	14.4	14.4	12.5	15.5	29.6	100						
	TP0621	12.8	12.8	12.8	15.4	32.2	76.6	100					
**Subfamily III**	TP0011	26.0	26.0	14.5	21.1	11.9	12.1	10.5	100				
	TP0610	23.5	23.5	19.3	19.2	8.9	12.1	11.3	23.1	100			
	TP0897	8.4	8.4	12.1	7.7	3.3	2.7	2.6	8.6	8.9	100		
	TP1031	34.4	34.4	26.4	32.8	12.6	15.5	14.3	22.3	21.5	9.1	100	
	MOSP	14.3	14.3	14.3	10.0	4.1	4.5	4.1	7.8	9.9	11.3	9.9	100

**CT region**		**Subfamily I**				**Subfamily II**			**Subfamily III**				
		TP0117	TP0131	TP0316	TP0620	TP0313	TP0317	TP0621	TP0011	TP0897	TP0610	TP1031	MOSP
**Subfamily I**	TP0117	100											
	TP0131	100	100										
	TP0316												
	TP0620	92.5	92.5		100								
**Subfamily II**	TP0313	65.9	65.9		63.5	100							
	TP0317	65.9	65.9		63.5	100	100						
	TP0621	66.3	66.3		63.9	93.6	93.6	100					
**Subfamily III**	TP0011	50.9	50.9		49.6	48.0	48.0	48.4	100				
	TP0610	66.5	66.5		66.5	59.2	59.2	62.1	51.8	100			
	TP0897	44.3	44.3		44.8	45.9	45.9	45.4	40.4	48.5	100		
	TP1031	77.6	77.6		73.6	62.1	62.1	62.1	51.8	66.7	47.8	100	
	MOSP	33.3	33.3		33.3	29.4	29.4	30.3	28.3	31.8	32.6	36.8	100

Table 7: Sequence similarity of *T. pallidum* efflux OMFs

% Sequence Similarity

	TP0966	TP0967	TP0968	TP0969
**TP0966**	100			
**TP0967**	39.1	100		
**TP0968**	33.6	31.6	100	
**TP0969**	29.9	34.1	30.3	100

Table S8: RMSD values of *T. pallidum* OMFs against *E. coli* TolC

Trimeric Model	RMSD (Å)
TP0966	0.73
TP0967	0.72
TP0968	0.41
TP0969	0.71

Table S9: Predicted BCEs of *T. pallidum* efflux OMFs

TP0966

BCE	Region	Residue	Score
BCE1	ECL1	THR173	-3.476
BCE1	ECL1	TYR174	-2.812
BCE1	ECL1	GLN175	-2.502
BCE1	ECL1	LEU177	-3.695
BCE2	loop of periplasmic tip	MET270	-2.509
BCE2	loop of periplasmic tip	ARG271	-3.139
BCE2	loop of periplasmic tip	ILE272	-2.52
BCE2	loop of periplasmic tip	GLN273	-1.152
BCE2	loop of periplasmic tip	ARG274	0.352
BCE2	loop of periplasmic tip	TYR275	-1.125
BCE2	loop of periplasmic tip	ALA277	-1.182
BCE2	loop of periplasmic tip	HIS278	-3.631
BCE2	loop of periplasmic tip	SER279	-2.04
BCE3	ECL2	SER387	-2.032
BCE3	ECL2	LYS389	-2.945
BCE3	ECL2	ARG390	-2.278
BCE3	ECL2	ILE391	-2.521
BCE4	loop of periplasmic tip	LYS496	-2.742
BCE4	loop of periplasmic tip	TRP497	-3.503
BCE4	loop of periplasmic tip	GLU499	-2.109
BCE4	loop of periplasmic tip	ARG500	-1.73
BCE4	loop of periplasmic tip	GLY501	-0.918
BCE4	loop of periplasmic tip	ALA502	-3.244

TP0967

BCE	Region	Residue	Score
BCE1	Periplasmic girdle	THR87	-3.292
BCE1	Periplasmic girdle	ILE88	-3.695
BCE1	Periplasmic girdle	GLN90	-2.668
BCE1	Periplasmic girdle	SER91	-2.518
BCE1	Periplasmic girdle	GLN92	-3.347
BCE1	Periplasmic girdle	TYR93	-2.329
BCE1	Periplasmic girdle	ASP94	-2.297
BCE1	Periplasmic girdle	LYS95	-2.766
BCE1	Periplasmic girdle	GLN96	-3.407
BCE1	Periplasmic girdle	ARG97	-3.124
BCE2	ECL2	ASN148	-3.302
BCE2	ECL2	GLY149	-3.446
BCE2	ECL2	GLY152	-3.175
BCE3	Periplasmic girdle	HIS183	-2.051
BCE3	Periplasmic girdle	VAL184	-3.547
BCE3	Periplasmic girdle	ALA186	-2.793
BCE3	Periplasmic girdle	VAL187	-2.899
BCE3	Periplasmic girdle	ASN188	-3.108
BCE3	Periplasmic girdle	THR189	-2.345
BCE3	Periplasmic girdle	LYS190	-2.44
BCE3	Periplasmic girdle	TYR191	-2.129
BCE3	Periplasmic girdle	GLU192	-2.424
BCE3	Periplasmic girdle	ALA193	-2.204
BCE3	Periplasmic girdle	LEU194	-3.698
BCE4	Periplasmic girdle	GLU333	-3.573
BCE4	Periplasmic girdle	ALA334	-3.268
BCE4	Periplasmic girdle	ALA335	-3.193
BCE4	Periplasmic girdle	ASP336	-1.302
BCE4	Periplasmic girdle	ASN337	-2.277
BCE4	Periplasmic girdle	GLU338	-1.432
BCE4	Periplasmic girdle	ARG339	-0.308
BCE4	Periplasmic girdle	GLU340	-0.901
BCE4	Periplasmic girdle	GLU341	-0.509
BCE4	Periplasmic girdle	ARG342	0.157
BCE4	Periplasmic girdle	ALA343	-0.092
BCE4	Periplasmic girdle	VAL344	-2.626
BCE5	Periplasmic girdle	ALA404	-2.928
BCE5	Periplasmic girdle	HIS405	-0.891
BCE5	Periplasmic girdle	LEU406	-0.824
BCE5	Periplasmic girdle	ARG407	0.13
BCE5	Periplasmic girdle	GLY408	0.512
BCE5	Periplasmic girdle	LYS409	-0.901
BCE5	Periplasmic girdle	ASN410	-1.233
BCE5	Periplasmic girdle	GLN411	-0.73
BCE5	Periplasmic girdle	ARG412	0.861
BCE5	Periplasmic girdle	LEU413	-2.053
BCE5	Periplasmic girdle	HIS414	-2.804
BCE5	Periplasmic girdle	ASP415	-0.951
BCE5	Periplasmic girdle	ALA416	-2.145
BCE5	Periplasmic girdle	ALA419	-3.123

TP0968

BCE	Region	Residue	Score
BCE1	ECL2	ILE140	-3.178
BCE1	ECL2	GLY141	-1.591
BCE1	ECL2	VAL142	-1.714
BCE1	ECL2	ARG143	-2.393
BCE1	ECL2	SER144	-2.327
BCE1	ECL2	PRO145	-3.319
BCE1	ECL2	ARG146	-2.043
BCE1	ECL2	TRY147	-1.786
BCE1	ECL2	ASN148	-0.227
BCE1	ECL2	ASN149	-0.189
BCE1	ECL2	LEU150	-0.978
BCE1	ECL2	VAL151	-1.987
BCE1	ECL2	VAL152	-1.193
BCE1	ECL2	SER153	-2.983
BCE1	ECL2	ILE154	-2.278

TP0969

BCE	Region	Residue	Score
BCE1	Periplasmic girdle	GLU63	-3.518
BCE1	Periplasmic girdle	ALA64	-2.417
BCE1	Periplasmic girdle	HIS66	-2.106
BCE1	Periplasmic girdle	ASN67	-0.263
BCE1	Periplasmic girdle	GLU68	-0.421
BCE1	Periplasmic girdle	VAL69	-0.796
BCE1	Periplasmic girdle	ARG70	1.594
BCE1	Periplasmic girdle	THR71	-0.516
BCE1	Periplasmic girdle	LYS72	-1.35
BCE1	Periplasmic girdle	THR73	-2.123
BCE2	ECL1	GLY93	-1.216
BCE2	ECL1	ALA94	-3.358
BCE2	ECL1	ASN95	0.984
BCE2	ECL1	GLY96	-1.615
BCE2	ECL1	TRY97	-1.6691
BCE2	ECL1	LYS98	-2.954
BCE2	ECL1	GLU100	-2.689
BCE3	Periplasmic girdle	SER144	-3.574
BCE3	Periplasmic girdle	SER145	-0.756
BCE3	Periplasmic girdle	VAL146	-0.544
BCE3	Periplasmic girdle	ARG147	-0.398
BCE3	Periplasmic girdle	PRO148	-0.232
BCE3	Periplasmic girdle	THR149	0.2
BCE3	Periplasmic girdle	LEU150	1.366
BCE3	Periplasmic girdle	LEU151	-1.79
BCE3	Periplasmic girdle	ASN152	-0.198
BCE3	Periplasmic girdle	SER153	-1.118
BCE3	Periplasmic girdle	LEU154	-1.131
BCE3	Periplasmic girdle	LYS155	-1.254
BCE3	Periplasmic girdle	GLU156	-2.654
BCE3	Periplasmic girdle	LYS157	-2.368
BCE3	Periplasmic girdle	THR158	-1.103
BCE3	Periplasmic girdle	TYR159	-3.316
BCE3	Periplasmic girdle	GLN160	-3.334
BCE3	Periplasmic girdle	LYS161	-2.859
BCE4	loop of periplasmic tip	VAL210	-2.46
BCE4	loop of periplasmic tip	LYS211	-2.986
BCE4	loop of periplasmic tip	ARG212	-0.147
BCE4	loop of periplasmic tip	GLN213	1.466
BCE4	loop of periplasmic tip	GLY214	1.414
BCE4	loop of periplasmic tip	TRY215	2.15
BCE4	loop of periplasmic tip	GLN216	-0.525
BCE4	loop of periplasmic tip	ASP218	-2.799
BCE4	loop of periplasmic tip	SER219	-2.366
BCE5	Periplasmic girdle	ILE304	-3.573
BCE5	Periplasmic girdle	GLN306	-2.641
BCE5	Periplasmic girdle	ARG307	-1.433
BCE5	Periplasmic girdle	GLY308	-1.016
BCE5	Periplasmic girdle	THR309	-1.05
BCE5	Periplasmic girdle	ASP310	-2.059
BCE5	Periplasmic girdle	TYR311	-1.798
BCE5	Periplasmic girdle	SER312	-1.108
BCE5	Periplasmic girdle	PHE314	-3.605
BCE6	ECL2	THR345	-2.801
BCE6	ECL2	TYR346	-3.547
BCE6	ECL2	GLY347	-1.016
BCE6	ECL2	GLY348	-0.208
BCE6	ECL2	THR349	-2.36
BCE6	ECL2	ASN351	-3.123
BCE6	ECL2	MET352	-3.36
BCE6	ECL2	PHE354	-2.919
BCE6	ECL2	GLY356	-2.105
BCE6	ECL2	GLY357	-2.675
BCE6	ECL2	ASP358	-2.722
BCE6	ECL2	SER360	-3.412
BCE6	ECL2	PHE361	-3.632
BCE6	ECL2	VAL363	-3.687

Table S10. Primers

Primer name	Description	5' to 3' Sequence
MOSP^N domain of *tp0897*-F	Amplification of *tprK* N-terminal domain	AGCCATATGGCTAGCCAAGTGAGCTTCACCCCGG
MOSP^N domain of *tp0897*-R	Amplification of *tprK* N-terminal domain	GTGGTGGTGGTGGTGACCCGCCGCCAGCGCACG
IFPC-pET28Vector-F	Amplification of pET28a	CACCACCACCACCACCAC
IFPC-pET28Vector-R	Amplification of pET28a	GCTAGCCATATGGCTGCCG
tp0856-F	RT-PCR of *tp0856*	TGAGTGTTCTGGACGGTTCG
tp0856-R	RT-PCR of *tp0856*	GACGCACAGTGAGCTAACCT
tp0856-*tp0858*-F	RT-PCR of *tp0856*-*tp0858* intergenic	ATGTCGCTCGGTGCCTCTAT
tp0856-*tp0858*-R	RT-PCR of *tp0856*-*tp0858* intergenic	GCGTGCATTACAATGGGCAC
tp0858-F	RT-PCR of *tp0858*	GGGAGCTGGCTTTCTTCCAT
tp0858-R	RT-PCR of *tp0858*	TTTTCTACCACCACGGAGCC
tp0858-*tp0859*-F	RT-PCR of *tp0858*-*tp0859* intergenic	AGGCTTCGCACTACCTTTCC
tp0858-*tp0859*-R	RT-PCR of *tp0858*-*tp0859* intergenic	TTGGTGTTCGGTTTTGCACG
tp0859-F	RT-PCR of *tp0859*	CATGTCCAGCAAAACCACCG
tp0859-R	RT-PCR of *tp0859*	GTCAAAGAGCTCCAACGGGA

References:

1. LaFond RE, Centurion-Lara A, Godornes C, Van Voorhis WC, Lukehart SA. 2006. TprK sequence diversity accumulates during infection of rabbits with *Treponema pallidum* subsp. *pallidum* Nichols strain. Infect Immun 74:1896-906.
2. Kringelum JV, Lundegaard C, Lund O, Nielsen M. 2012. Reliable B cell epitope predictions: impacts of method development and improved benchmarking. PLoS Comput Biol 8:e1002829.
3. Nishikawa K, Ooi T. 1980. Prediction of the surface-interior diagram of globular proteins by an empirical method. Int J Pept Protein Res 16:19-32.
